# Metabolic markers detect early ostedifferentiation of mesenchymal stem cells from multiple donors

**DOI:** 10.1186/s13287-025-04419-x

**Published:** 2025-06-07

**Authors:** Daniela S. C. Bispo, Inês C. R. Graça, Catarina S. H. Jesus, João E. Rodrigues, Marlene C. Correia, Sabrina Atella, Iola F. Duarte, Brian J. Goodfellow, Mariana B. Oliveira, João F. Mano, Ana M. Gil

**Affiliations:** Department of Chemistry, CICECO - Aveiro Institute of Materials, University of Aveiro, Campus Universitario de Santiago, Aveiro, 3810-193 Portugal

**Keywords:** Osteogenic differentiation, Osteogenesis, Bone regeneration, Mesenchymal stem cells, Metabolic markers, Metabolomics, Nuclear magnetic resonance (NMR) spectroscopy

## Abstract

**Background:**

Mesenchymal stem cells (MSC) are pivotal bioengineering tools, offering significant promise for applications in bone regeneration. However, their therapeutic potential is limited by inter-donor variability and experimental issues. This study aimed to identify robust metabolic markers of osteodifferentiation applicable across multiple donors, while providing insight into the metabolic pathways actively involved in the process.

**Methods:**

Untargeted nuclear magnetic resonance (NMR) metabolomics was applied to characterize the intra- and extracellular metabolic adaptations of human adipose-derived MSC (hAMSC) undergoing osteogenic differentiation, compared to proliferation alone. Multivariate and univariate statistical analysis was carried out on data from three independent donors, and cross-validation was employed to evaluate the predictive capacity of the proposed markers.

**Results:**

Variations in the levels of selected (nine) intracellular and (seventeen) extracellular metabolites detect osteodifferentiation by day 7 (out of 21), with nearly 100% accuracy. These signatures suggest a metabolic shift from glycolysis/OxPhos to lactic fermentation, fatty acid *β-*oxidation and phosphocreatine hydrolysis. Intracellular glucose, lactate, citrate and specific amino acids are redirected towards protein synthesis and glycosylation, with some of the secreted metabolites (*e.g.*, citrate) seemingly involved in biomineralization and other extracellular roles. Membrane metabolism, antioxidant mechanisms and adenosine metabolism are also impacted by osteodifferentiation.

**Conclusions:**

These findings reveal effective donor-independent markers of hAMSC osteodifferentiation, with a robust extracellular signature standing out for potential rapid and non-invasive detection of osteocommitted cells.

**Supplementary Information:**

The online version contains supplementary material available at 10.1186/s13287-025-04419-x.

## Graphical Abstract

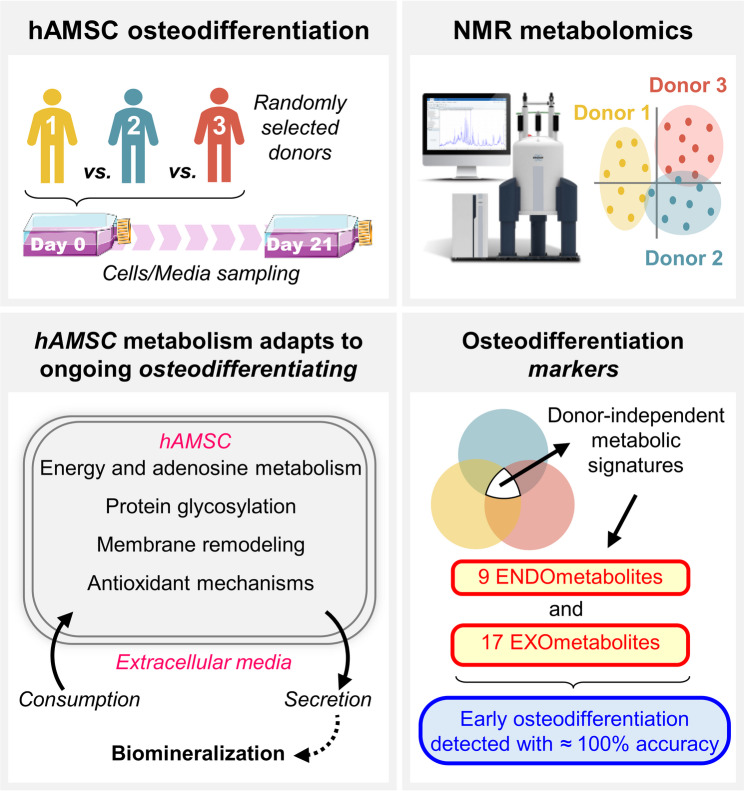

## Introduction

Mesenchymal stem cells (MSC) are pivotal bioengineering tools that can be used effectively in tissue regeneration [1]. However, their inherent biological variability due to inter-donor and tissue source heterogeneity often limits therapeutic applications [2]. In addition, the lack of standardized user-independent protocols for MSC handling also contributes to this heterogeneity [3]. Although existing assays are invaluable for detecting well-known gene/protein markers, they are often time-consuming, user-dependent and prone to reproducibility issues [4, 5]. The resulting high variability in MSC behavior and differentiation performance calls for improved characterization guidelines, ideally based on new robust markers [6]. Increasing interest has therefore arisen in the development of new strategies to unambiguously monitor and predict MSC behavior, for an effective selection of donors/cells with superior therapeutic quality [7]. Pre-screening donors according to their characteristics (age, gender, others) could be beneficial [8], however that would entail time consuming characterization of sufficiently large donor cohorts. Alternatively, the identification of donor-independent markers may be a more practical approach.

Within the scope of MSC osteogenic differentiation (typically 21 days), efforts to effectively detect and predict osteocommitment have explored cell morphology [9–12] and motility [13], electrochemical properties [14, 15], and biochemical adaptations expressed by genes [16–18], proteins [19, 20] or metabolites [21, 22]. However, to the best of our knowledge, adequate statistical validation of proposed markers remains limited, having mostly addressed morphological data [9–12] and specific proteins/genes [16, 17, 20]. In particular, a large set of human MSC (hMSC) morphological features (*e.g.* fiber breadth, fiber length, hole area, among others), analyzed by ridge regression-based machine learning, was used to predict alkaline phosphatase (ALP) levels at day 14 of osteodifferentiation (correlation coefficient > 0.90) [9]. Linear discriminant analysis of early hMSC morphological features revealed the differential minor axis length of cells as a predictor of late osteodifferentiation by day 3 (> 90% accuracy), outperforming traditional biochemical assays [10]. Regarding end lineage recognition, multidimensional scaling analysis of 43 cytoskeleton-based descriptors (based on actin shape, staining intensity, texture and spatial distribution) effectively distinguished osteogenic from adipogenic hMSC, within 3–4 days of differentiation (> 83% accuracy) [11]. More recently, convolutional neural networks, pre-trained on a range of morphological features, were able to distinguish MSC osteodifferentiation from proliferating and adipogenic cells with over 94% accuracy [12]. Omics-derived data have also been increasingly recognized as highly promising in this context. An initial study of human bone marrow MSC (BMMSC) [20] reported 8 intracellular phosphoproteins able to predict mineralization outcomes upon 7 days of differentiation, with nearly 100% accuracy. Subsequent receiver operating characteristic (ROC) analysis of differential gene expression data identified the enzymes SAM and HD domain-containing deoxynucleoside triphosphate triphosphohydrolase (SAMHD1), and FK506 binding protein prolyl isomerase 5 (FKBP5) as promising osteodifferentiation biomarkers (area under curve (AUC) > 0.8) [17]. Furthermore, a model integrating open-access transcriptomic data with machine learning algorithms was able to predict lineage fate direction (osteogenic, chondrogenic and adipogenic) with 90% accuracy, within the first 3 days of differentiation [16]. Many of the above studies have considered the average behavior of multiple MSC donors [9–12] or public datasets [16, 17], to mitigate biases linked to individual patients. Some reports have also addressed MSC metabolomics data, mainly to: (i) profile the endometabolome of different cell types/origins [23], (ii) identify the effects of donor characteristics, namely body mass index [24], gender [25] and age [26], and (iii) measure metabolic adaptations to differentiation or other culture conditions [22, 27]. The relevance of metabolomics in stem cell research has been reviewed recently [28], and both intracellular [21, 27, 29] and extracellular [22, 30, 31] metabolomes have been shown to respond to osteodifferentiation, their articulated interpretation having been attempted [31, 32]. In particular, osteodifferentiation has been suggested to impact energy metabolism and pathways involving lipids, specific amino acids (AA), antioxidative mechanisms and aminoacyl-tRNA biosynthesis. A preliminary proposal of possible intracellular metabolic markers has been suggested for osteodifferentiating human adipose-derived MSC (hAMSC) from 2 independent donors [21]. This is especially significant within the search for viable alternatives to BMMSC, the clinical applicability of which may be constrained by invasive harvesting and limited availability [33]. Notably, hAMSC display robust osteogenic capacity (albeit at a slower pace than BMMSC), while offering improved accessibility, higher proliferative potential, and potentially greater resilience to aging/senescence.

In this work, the intra- and extracellular metabolic features accompanying the osteodifferentiation of hAMSC are characterized for 3 independent donors, over a period of 21 days and compared to proliferating cells. By employing untargeted nuclear magnetic resonance (NMR) metabolomics we establish, for the first time to our knowledge, potentially reliable donor-independent metabolic signatures (sets of metabolite variations) that may serve as predictive markers of osteodifferentiation, while elucidating particularly responsive metabolic pathways accompanying the process. The novel integration of endo- and exometabolomic adaptations facilitates the development of rapid and non-invasive strategies to detect and predict early MSC osteodifferentiation.

## Materials and methods

### Isolation and expansion of hAMSC

Human AMSC were sourced from 3 randomly selected healthy donors (numbered 1 to 3). Adipose tissue from donor 1 (unavailable donor characteristics) was obtained *via* abdominoplasty, under an agreement between the University of Aveiro and “Hospital da Luz”, Aveiro, dated 17th February 2023, and processed accordingly to protocols mentioned previously (21). Cells from donors 2 (27-year-old African American female) and 3 (42-year-old Caucasian female), obtained through breast reduction and liposuction procedures respectively, were purchased from American Type Culture Collection (Lot 70017032, Ref. ATCC PCS-500-011) and Lonza (Lot 22TL018258, Ref. LSLZPT-5006). For all donors, cryopreserved hAMSC were thawed, plated in culture flasks (T175), expanded in minimum essential alpha medium (*α*-MEM, Gibco™ 12000063, Waltham, MA, USA) supplemented with 10% v/v heat-inactivated fetal bovine serum (FBS, Gibco 10270106) and 1% v/v antibiotics (penicillin − streptomycin, Gibco 15240062) at 37 °C in a humidified 5% CO_2_ incubator and passaged as described previously [27, 31].

### Osteogenic differentiation and sampling of hAMSC

**Fig. 1:**
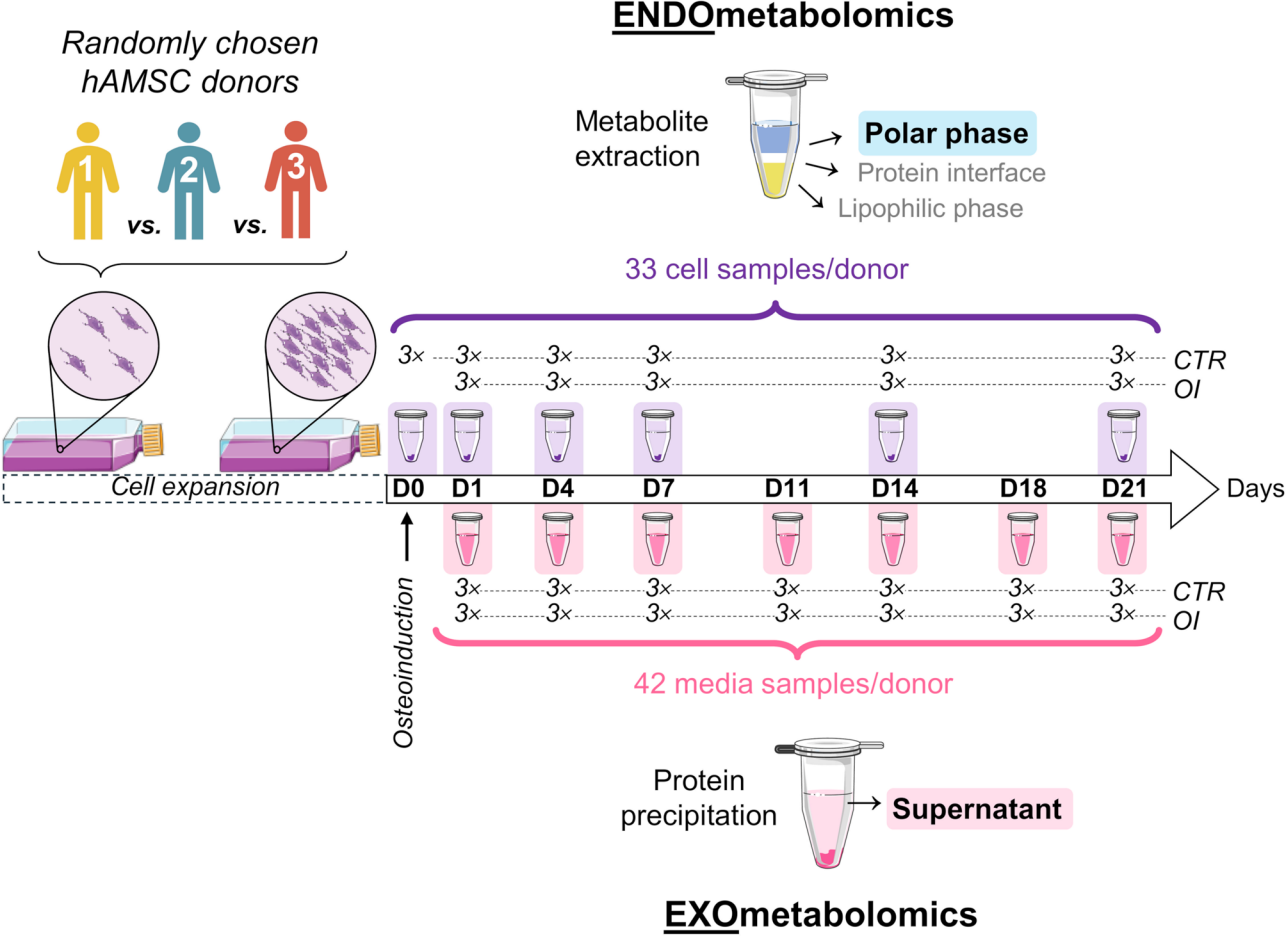
Experimental design for hAMSC harvesting and media sampling (from 3 donors) throughout proliferation (control) and osteodifferentiation. For each donor, cell samples were collected (in triplicate) on D0, D1, D4, D7, D14 and D21 (purple) and media samples (also in triplicate) on D1, D4, D7, D11, D14, D18 and D21 (pink). Some figure elements were adapted from Servier Medical Art and licensed under a Creative Commons Attribution 3.0 Unported (CC BY 3.0) license

Osteodifferentiation of hAMSC was induced at passages 5 or 6, for 21 days, in T175 culture flasks (for metabolomics) and in 48-well plates (for biochemical assays). An independent experiment was conducted for each of the donors (Fig. 1). For metabolomics, cells were seeded at a 0.5 × 10^6^ cells/flask density. After reaching 100% confluence, day 0 (D0) cell samples were collected in triplicate from independent flasks, and media were replaced in the remaining flasks as follows: 15 control (CTR) flasks in standard growth medium and 15 osteoinduced (OI) flasks in medium supplemented with 10 mM *β-*glycerophosphate (*β-*GP, Sigma-Aldrich G9422), 50 µg/mL L-ascorbic acid (Sigma A0278) and 10 nM dexamethasone (ACROS Organics 230300010). Media were replaced 2× per week, on days (Di) 0, 4, 7, 11, 14, 18 and 21. Cells were trypsinized and collected in triplicate on D0, D1, D4, D7, D14 and D21 (Fig. 1). Cell suspensions were filtered through 100 μm pore strainers, centrifuged (300 g, 4 °C, 5 min) and rinsed twice in phosphate-buffered saline (PBS) solution. For intracellular metabolomics (endometabolomics), cell numbers/sample were 3.8$\:-$21.1 × 10^6^, 2.3$\:-$8.6 × 10^6^ and 1.4$\:-$9.1 × 10^6^ for donors 1, 2 and 3, respectively. For extracellular metabolomics (exometabolomics), media samples were collected on D1, D4, D7, D11, D14, D18 and D21 (days when cell collection and/or medium exchange was carried out) (Fig. 1) and filtered through 40 μm pore strainers to remove cellular debris. Due to contamination and/or technical issues, triplicate samples could not always be retrieved leaving the following sample groups as follows: donor 1: CTR D0 cells *n =* 2, OI D7 media *n =* 0; donor 2: CTR D7,D18 media *n =* 2, OI D11,D21 media *n =* 1-2, OI D21 cells *n =* 1). This fact is consistently indicated in the relevant data representations having, however, not compromised the end results. For biochemical assays, cell samples were rinsed 2× with PBS and lysed by osmotic/thermal shock. Collected media and cell samples were stored at − 80 °C.

### Expansion of human primary osteoblasts

Cryopreserved Clonetics™ normal human osteoblasts (NHOst) from a healthy 26-year-old Caucasian male (Lonza, CC-2538, Lot 19TL217387) were subcultured using ReagentPack™ subculture reagents (Lonza, CC-5034) up to passage 5. Cells were maintained in Clonetics™ OGM™ Osteoblast media (Lonza, CC-3208) with OGM SingleQuot Kit supplements and growth factors (Lonza, CC-4193) for either 6 days (100% confluence) or 13 days, followed by cell collection.

### Biochemical assays

To evaluate cell proliferation, double-stranded DNA (dsDNA) was quantified for cell lysates (Quant-iT PicoGreen dsDNA assay kit, Molecular Probes, Invitrogen) as previously described [21]. Osteodifferentiation was assessed by quantification of ALP activity (*p*-nitrophenol assay) and calcium (QuantiChrom calcium assay kit, DICA-500, BioAssay Systems) in cell lysates, as well as OCN (SimpleStep ELISA kit for human OCN, ab270202, Abcam) in media, following established protocols [21, 27, 31].

The corresponding results (Figure S1, Additional file 1) highlight the inherent variability in proliferation and osteodifferentiation capacities of hAMSC from different donors. Compared to donors 2 and 3, donor 1 displays higher cell proliferation rates (dsDNA levels, Figure S1A, Additional file 1) and higher extents of differentiation and mineralization. The latter processes are expressed by more elevated levels of ALP and, to a lesser extent, OCN, expected after D14 [34], as well as by higher Ca^2+^ levels at D21 (Figure S1B-D, Additional file 1). The atypically high Ca^2+^ levels at D7 seen for both proliferating and osteogenic cells may reflect previously reported fluctuations in Ca^2+^ concentrations, in hAMSC [35, 36].

### Sample preparation for NMR spectroscopy

Intracellular metabolites (endometabolites) were extracted using a methanol-chlorofor*m-*water method, as described elsewhere [21]. Briefly, cell pellets were re-suspended in 1 mL of a cold solution of methanol (Honeywell Riedel-de-Haen 14262) and Milli-Q water (in a 4:1 ratio), transferred to glass tubes containing 150 mg of glass beads (ø = 0.5 mm), and vortexed for 2 min at room temperature (RT 25 °C). Cold chloroform (400 µL, Honeywell Riedel-de-Haen 650471) was then added and vortexed for 2 min at RT, followed by 400 µL of cold chloroform plus 360 µL of cold Milli-Q water with further vortexing for 2 min at RT. After incubating at − 20 °C for 10 min, samples were centrifuged (2000 g, 20 min, RT) and the upper polar phases were collected, dried and stored at − 80 °C until analysis.

Extracellular metabolites (exometabolites) were measured in cell media, upon protein-precipitation of blank and conditioned media [31]. Briefly, 600 µL of 100% methanol at– 80 °C were added to microcentrifuge tubes containing 300 µL of medium sample. After incubating at– 20 °C for 30 min, samples were centrifuged (13000 g, 20 min, RT), the supernatant was collected, dried under vacuum and stored at– 80 °C. To prepare samples for NMR analysis, cellular polar extracts were re-suspended in 625 µL of 100 mM phosphate buffer (pH 7.4), in D2O (99.9% deuterium, Eurisotop D216) containing 0.1 mM 3-(trimethylsilyl)-propionic-2,2,3,3-d4 acid (TSP, in D2O, Sigma-Aldrich 293040) for chemical shift referencing. For exometabolome analysis, dry media samples were resuspended in 700 µL of the same phosphate buffer, centrifuged (13000 g, 5 min, RT) and the supernatant was collected. A volume of 550 µL from each sample (pH adjusted to 7.4) was transferred into a 5 mm NMR tube.

### NMR spectroscopy

Standard unidimensional ^1^H NMR spectra were acquired with water presaturation (*noesypr1d* pulse sequence, Bruker library) on a Bruker Avance III spectrometer operating at 500.13 MHz for proton (at 298 K). Addition of 512 transients (endometabolome) or 256 transients (exometabolome) into 32 k data points was carried out, using a spectral width of 7002.801 Hz, an acquisition time of 2.3 s and a relaxation delay (d1) of 4 s. Prior to Fourier transformation, each free induction decay was zero-filled to 64 k data points (endometabolome) or 128 k data points (exometabolome) and multiplied by a 0.3 Hz exponential line-broadening function. Subsequently, spectra were manually phased, baseline corrected and chemical shift referenced internally to TSP at *δ* 0.00. Peaks were assigned with the aid of two-dimensional ^1^H-^1^H total correlation (TOCSY) and ^1^H-^13^C heteronuclear single quantum correlation (HSQC) spectra, spiking experiments, the literature and spectral databases, such as the Bruker BBIOREFCODE AMIX database, the human metabolome database (HMDB) and Chenomx NMR Suite (Chenomx Inc., Edmonton, AB, Canada).

### Statistical analysis

Multivariate analysis (MVA) included principal component analysis (PCA) and partial-least-squares discriminant analysis (PLS-DA) (SIMCA-P 11.5, Umetrics, Umeå, Sweden) were used on either full resolution ^1^H NMR spectra or selected signal integrals. Spectral regions for water (*δ* 5.2–4.5) and TSP (*δ* 0.6 − 0.0) were excluded. Other excluded signals comprised methanol (*δ* 3.3 − 3.4, extraction solvent), ethanol (*δ* 1.1 − 1.2, 3.6 − 3.7, cleaning solvent), *β-*GP and glycerol (*δ* 3.5 − 3.9, 4.1–4.2, osteogenic supplement and hydrolysis product in media), and dimethylsulfoxide (*δ* 2.8–2.7, dexamethasone solvent in media). Prior to MVA, ^1^H NMR spectra of the polar extracts were aligned using recursive segment-wise peak alignment (Matlab R2014a, The MathWorks Inc., Natick, MA, USA) and normalised to total spectral area, to minimize chemical shift variations and differences in sample cell numbers, respectively. For each cell media replicate j (of three replicates), collected at day i, each spectral data point, S_i, j_, was corrected to account for media exchanges and different cell numbers (S_i, j corr_). This was carried out using expression 1, where $$\:\stackrel{-}{\text{S}}$$_i−1 corr_ is the mean of the corrected data points, for all replicates, at the previous time point (set to zero for S_1,j corr_); $$\:\stackrel{-}{\text{B}}{\:}_{\text{i}-1}$$$$\:{\text{S}}_{\text{i}-1,\text{j}}$$ and $$\:{\text{S}}_{\text{i},\text{j}}$$); and $$\:{\text{T}\text{A}}_{\text{i},\text{j}}$$ is the total spectral area of each replicate j, at day i.

1 $${{\rm{S}}_{{\rm{i}},{\rm{j corr}}}} = {\overline S _{i - 1{\rm{ }}corr}} + \left( {\frac{{{{\rm{S}}_{{\rm{i}},{\rm{j}}}} - {{\overline B }_{{\rm{i}} - 1}}}}{{{\rm{T}}{{\rm{A}}_{{\rm{i}},{\rm{j}}}}}}} \right)$$

PCA and PLS-DA were performed after unit variance (UV) or centered data scaling. PLS-DA loading weights were back-transformed by multiplying each variable by its standard deviation and colored according to variable importance to the projection (VIP) (Matlab R2012a). Signals were integrated in the spectra (Amix 3.9.15, Bruker BioSpin, Rheinstetten, Germany). Integrals were normalized to spectral total area or using expression 1, for endometabolites and exometabolites, respectively. Relevant metabolite variations were selected based on their statistical significance (*p*-values < 0.05, Wilcoxon rank-sum nonparametric test) and spectral visual confirmation. Metabolite correlations were calculated through Spearman’s rank correlation coefficients (*ρ*), statistical tests and heatmaps generated using Python 3.6.5. For donor 3 (for which extra dsDNA sampling was performed for the same samples used for metabolomics), absolute concentrations were estimated (Chenomx NMR Suite). Enrichment pathway analysis was based on human metabolic pathways (Kyoto Encyclopedia of Genes and Genomes) (MetaboAnalyst 6.0.) using the normalized integrals of endometabolites. The robustness of PLS-DA models was assessed by Monte Carlo cross-validation (MCCV) (7 blocks and 500 iterations, in house software) and model performance evaluated by predictive power (Q^2^ values), sensitivity (sens), specificity (spec), and classification rate (CR, or accuracy). Metabolite contributions for sample group separation were evaluated using VIP values calculated with MetaboAnalyst 6.0.

## Results

### hAMSC proliferation alone is accompanied by donor-independent metabolic adaptations

**Fig. 2:**
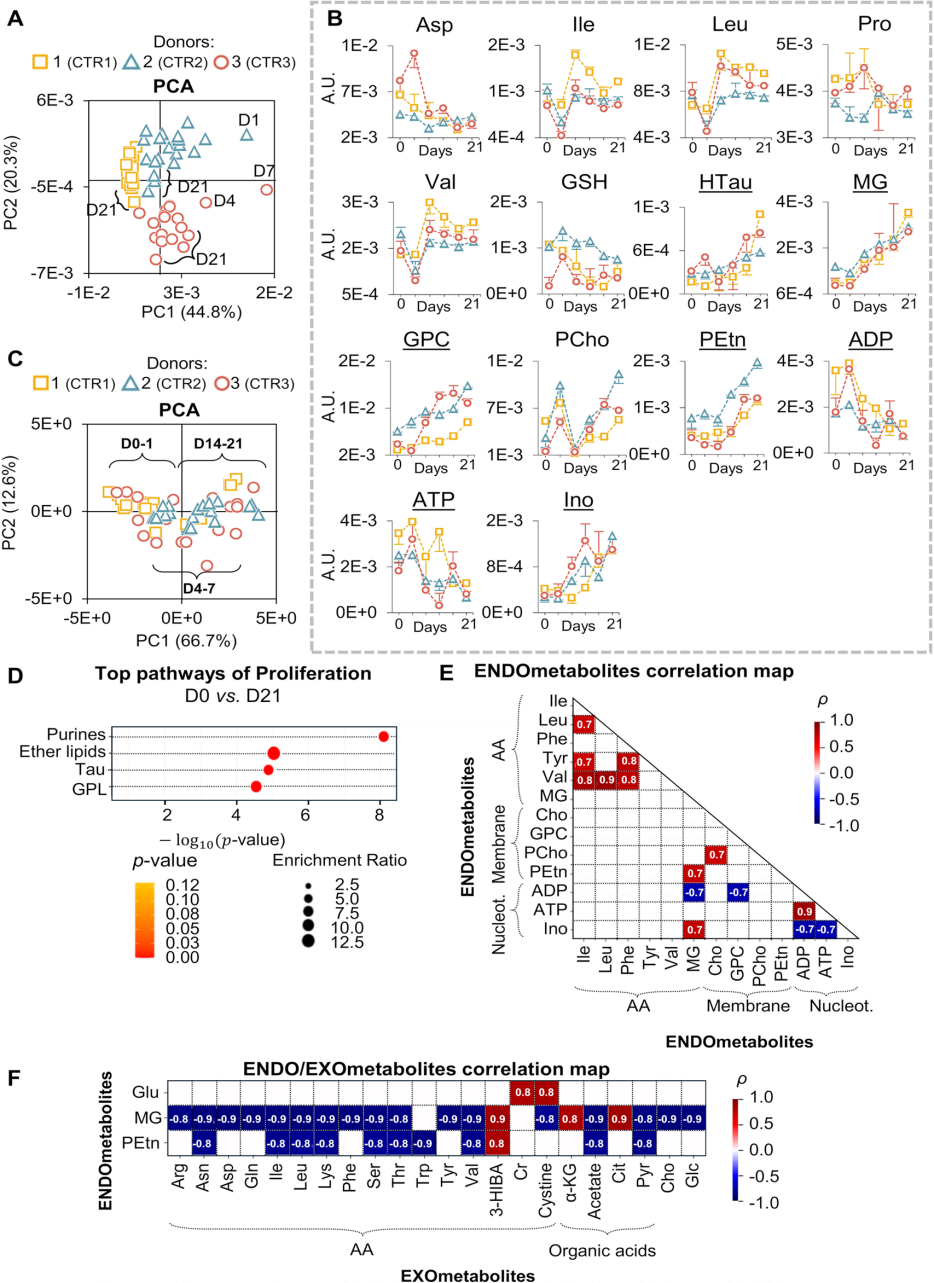
Metabolic changes in proliferating (non-differentiating) hAMSC (control, CTR). (**A**) PCA scores scatter plot of full NMR spectra of hAMSC polar extracts. (**B**) Time course evolution of the 14 endometabolites comprised in the donor-independent signature of proliferation (% variation in Table S2, Additional file 1). (**C**) PCA scores scatter plot of integrals of 7 selected endometabolites: HTau, MG, GPC, PEtn, Ino, ADP and ATP. (**D**) Top enriched pathways descriptive of hAMSC proliferation for all donors. (**E**) Endometabolite correlations, all timepoints (*ρ* >|0.7|, *p*-value < 0.001). (**F**) Endometabolite/Exometabolite correlations (*ρ* >|0.8|, *p*-value < 0.001). 3-HIBA, 3-hydroxyisobutyrate; ADP, adenosine diphosphate; ATP, adenosine triphosphate; Cho, choline; Cr, creatine; GPC, glycerophosphocholine; GPL, glycerophospholipids; GSH, glutathione (reduced); HTau, hypotaurine; Ino, inosine; MG, methylguanidine; PCho, phosphocholine; PEtn, phosphoethanolamine; Pyr, pyruvate; *α*-KG, *α*-ketoglutarate

Under control conditions, the ^1^H NMR spectra of hAMSC polar extracts reflect intracellular metabolic features accompanying cell proliferation (spectrum shown for donor 1 in Figure S2A, Additional file 1). Signals arise from just over 50 identified endometabolites (Table S1, Additional file 1), comprising 22 AA and derivatives, 12 nucleotides and derivatives, 7 organic acids, 5 membrane precursors, and 5 other metabolites (acetone, glucose (Glc), glycerol, *m-*inositol (*m-*Ino) and propylene glycol (possible contaminant)) (Table S1, Additional file 1). All of the above have been reported previously for hAMSC [27], except for hypotaurine (HTau) and 2-hydroxyisocaproate (2-HIC), to the best of our knowledge. Here, we examine how hAMSC metabolic profiles evolve during 21 days of proliferation alone, as this information is crucial for the identification of specific markers of osteodifferentiation. The grouping of each donor samples seen by PCA of the full-resolution NMR spectra (Fig. 2A) shows that inherent hAMSC intracellular profiles differ significantly between donors, with no clear dependence on culture time. Systematic pairwise PLS-DA between donors (Figure S3) and visual spectral inspection revealed lower glycerol and glycerophosphocholine (GPC) levels and higher formate content for donor 1, higher levels of creatine (Cr), taurine (Tau), acetate and acetone for donor 2, and higher levels of 2-HIC, lactate (Lac), Gly and Glu, along with lower Cr levels for donor 3. Hence, changes in these endometabolites must be interpreted with care, as they seem particularly sensitive to donor characteristics. Nevertheless, within the ca. 30 endometabolites varying during proliferation, 14 metabolites showed similar progressions over time for all 3 donors (Fig. 2B). This 14-endometabolite signature comprises increases in the levels of HTau, methylguanidine (MG), GPC, phosphoethanolamine (PEtn) and inosine (Ino) (variations > 85%, Table S2, top); and decreases in the levels of adenosine diphosphate (ADP) and adenosine triphosphate (ATP) (> 55%, Table S2, top, Additional file 1), although with large error due to low signal intensity and maintaining ADP/ATP ratios constant. Absolute quantitation estimated for donor 3 positioned metabolite concentrations at the µM/µg dsDNA level (Table S2, top, Additional file 1) and confirmed % variations. Furthermore, converging levels of Asp, Pro and reduced glutathione (GSH) at D21 (Fig. 2B), and comparable evolutions of branched-chain AA (BCAA) (Ile, Leu, Val) and phosphocholine (PCho) for the 3 donors (Fig. 2B), suggest a similar use/regulation of these metabolites. The above set of 14 endometabolite variations is proposed as a donor-independent signature of hAMSC proliferation. Indeed, PCA considering only the main varying metabolites (HTau, MG, GPC, PEtn, Ino, ADP and ATP) (underlined in Fig. 2B) abolishes inter-donor differences and reveals a common time evolution (D14-21 samples in positive PC1, Fig. 2C). Enrichment pathway analysis (Fig. 2D) highlights adaptations in the metabolism of purines, ether lipids, Tau and glycerophospholipids (GPL). Intracellular correlations (Fig. 2E) reveal direct (positive) correlations between ADP and ATP (comparable decreasing rates) and within BCAA and aromatic AA (which share pathway characteristics). In addition, inverse (negative) correlations of ADP with Ino, GPC and MG are noted. A donor-independent extracellular signature of proliferation was also identified in the spectra of cell media (Figures S2B, C and S4). Except for MG and 2-hydroxyisobutyrate (2-HIBA), all exometabolites detected here have been reported previously in hAMSC media [31]. For all donors, proliferation was accompanied by (i) uptake of 14 AA, Cr, cystine, pyroglutamate (PyroGlu), 2-HIBA, acetate, pyruvate (Pyr), choline (Cho), Glc and *m-*ino; and (ii) secretion of 3-hydroxyisobutyrate (3-HIBA), 3-methyl-2-oxovalerate (3M2OV), 3-hydroxybutyrate (3-HBA), *α*-ketoglutarate (*α*-KG), citrate (Cit), formate and Lac (Figure S4, Additional file 1). Correlation between endo- and exometabolites (Fig. 2F) reveals that higher intracellular MG levels correlate inversely with extracellular levels of 12 of the uptaken AA, cystine, acetate, Pyr, Cho and Glc. Increasing MG levels also correlate directly with increasing secretion of 3-HIBA (byproduct of Val degradation) and tricarboxylic acid (TCA) cycle intermediates *α*-KG and Cit. Increased intracellular PEtn levels exhibit a similar correlation pattern to MG (Fig. 2F), while intracellular Glu (rough decreasing tendency after D1) correlates directly with extracellular levels of uptaken Cr and cystine.

### hAMSC osteodifferentiation triggers robust and osteospecific donor-independent metabolic changes

**Fig. 3:**
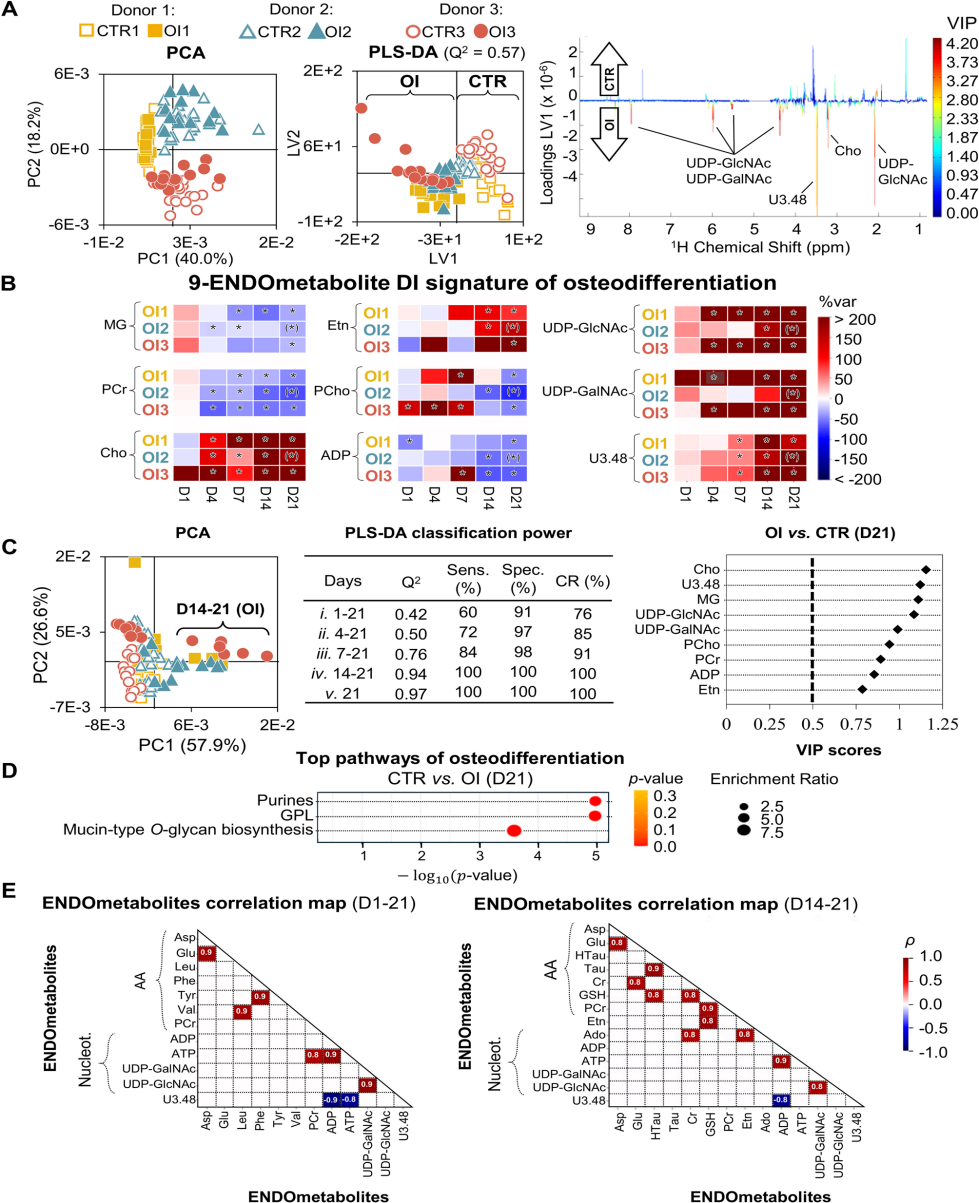
Changes in the endometabolome of osteodifferentiating hAMSC. (**A**) PCA scores scatter plot (left) obtained with full NMR spectra of hAMSC polar extracts, under control (CTR) and osteoinduced (OI) conditions, pairwise PLS-DA scores scatter plot (middle) comparing CTR to OI, and corresponding LV1 loadings plot (right). (**B**) Donor-independent (DI) osteogenic signature of 9 endometabolites (heatmap of % variation for OI compared to CTR at each timepoint), *, *p*-value < 0.05; ^(^*^)^, visually confirmed differences (*p*-value not calculated as *n* = 1 for D21 of OI2). (**C**) PCA scores scatter plot obtained with selected integrals (left), MCCV of PLS-DA models obtained excluding progressively earlier timepoints (middle), and VIP scores corresponding to D21 samples (model v.) (right). (**D**) Top enriched pathways related to osteodifferentiation at D21, compared to controls, considering all donors. (**E**) Spearman correlation maps for endometabolites across all timepoints (left) and in D14 and D21 (right), *ρ* >|0.8| and *p*-value < 0.001. Etn, ethanolamine; PCr, phosphocreatine; UDP-GalNAc, uridine diphospho-*N*-acetylgalactosamine; UDP-GlcNAc, uridine diphospho-*N*-acetylglucosamine; U*δ*, unassigned signal at chemical shift *δ*. Other metabolite abbreviations specified in the caption of Fig. 2. CR, Classification rate (accuracy); Q^2^, predictive power; sens., sensitivity; spec., specificity

When osteodifferentiating hAMSC samples are considered together with controls, PCA of the full NMR spectra of cell extracts continues to indicate differences mainly due to inherent inter-donor variability (Fig. 3A, left). However, pairwise PLS-DA unveils separation between controls (CTR) and differentiating cells (OI) for all 3 donors, with satisfactory robustness (Q^2^ 0.57) (Fig. 3A, middle). In agreement, the corresponding LV1 loadings (Fig. 3A, right) suggest that osteodifferentiation is generally accompanied by increases in Cho, uridine diphospho-*N*-acetylgalactosamine (UDP-GalNAc), uridine diphospho-*N*-acetylglucosamine (UDP-GlcNAc) and a metabolite with unassigned resonance at *δ* 3.48 (U3.48), as also suggested by visual spectral inspection. Pairwise inter-donor comparison through PLS-DA shows that model robustness is higher at later stages of differentiation (D7 to D21), for which Q^2^ values range from 0.93 to 0.71 (Figure S5), depending on the donor. A total of 37 metabolites are seen to vary in content during osteodifferentiation, compared to controls: 18 AA and derivatives, 7 nucleotides and derivatives, 5 membrane precursors, 3 organic acids and 4 other compounds (Glc, glycerol, *m-*Ino and acetone). Among these, 9 metabolites evolve similarly for all 3 donors, and distinctly from controls (Figure S6A, Additional file 1), therefore constituting a proposed donor-independent osteogenic-specific signature. This 9-metabolite signature is composed of marked increases in Cho, Etn, UDP-GlcNAc, UDP-GalNAc and U3.48 levels (> 80%, Table S2, bottom, Additional file 1), compared to controls and consistently with PLS-DA loadings (Fig. 3A, right).

Absolute concentrations estimated for some metabolites (based on dsDNA quantitation available for donor 3 samples) confirmed that both Cho and UDP-GlcNAc levels increase ca. 5-fold, to 1.6 and 6.2 µM/µg ds DNA, respectively (Table S2, Additional file 1). In addition, relatively lower levels are noted for MG (after D4), phosphocreatine (PCr, after D1), PCho (after D14), and ADP (after D7) (all in the sub-µM/µg ds DNA range, Table S2, Additional file 1). The magnitude of variation of these metabolites (heatmap representation chosen for the sake of clarity, Fig. 3B), compared to control cells, illustrates general inter-donor agreement emerging at D4-D7. Increased Cho/PCho and Etn/PEtn ratios, and decreased HTau/Tau ratios, were also found to be donor-independent (Figure S6B, Additional file 1). Additional metabolites varied consistently in 2 of the 3 donors, thus not strictly being part of a donor-independent signature but possibly also relating to osteodifferentiation (Figure S7A, Additional file 1). These included higher levels of Gln (peaking at D4-D7), GSH, adenosine (Ado), Ado/Ino and Cr/PCr ratios; and lower levels of HTau, AMP, Ino, PEtn and Lac. Intracellular levels of Asp, acetate, formate and ATP exhibited variations similar to controls, possibly mainly reflecting proliferation (Figure S7B, Additional file 1). The donor-independent 9-metabolite signature of osteodifferentiation identified above (↑ Cho, Etn, UDP-GlcNAc, UDP-GalNAc, U3.48; ↓ ADP, MG, PCho, PCr) can also be seen by PCA of the corresponding signal integrals (Fig. 3C, left), which abolishes donor separation and separates D14-D21 osteogenic samples in PC1. Sample positioning in PC1 may therefore serve as an indicator of osteodifferentiation progress. The classification power of PLS-DA models (CTR vs. OI) built for the 9 selected metabolites is high (Q^2^ 0.76–0.97) when including D7-D21 (iii. to v. in Fig. 3C, middle), with 100% sensitivity, specificity and accuracy from D14 onwards. At D21, Cho, MG, UDP-GlcNAc and U3.48 are particularly important for group separation, followed by UDP-GalNAc, PCho, PCr, ADP and Etn (Fig. 3C, right). In agreement, the main affected pathways are related to purines, GPL and *O*-glycan biosynthesis (Fig. 3D). Intracellular correlation analysis (Fig. 3E, left) reveals concomitant variations (direct correlations) for UDP-GlcNAc and UDP-GalNAc, ATP and PCr, and Glu and Asp. Also, inverse correlations are seen for U3.48 with ADP and ATP (Fig. 3E, left), in addition to features previously noted as descriptive of proliferation. When only D14-D21 are considered (Fig. 3E, right), positive correlations become visible for GSH (with HTau, Cr, PCr, Etn) and for Tau with HTau. We have further compared the intracellular spectral profile of differentiated hAMSC (D21) with that of primary NHOst osteoblasts. Conclusive analysis is hindered by the strong dependence of the latter on culture time (Figure S8, black and grey bars for 6 and 13 culture days, respectively). However, it is interesting to note that 23 of the metabolites present in differentiated hAMSC are absent in osteoblasts (Table S1, Additional file 1), these including part of the proposed osteogenic donor-independent signature (namely, MG, Etn, UDP-GlcNAc and UDP-GalNAc). In addition, the relative amounts of the remaining 5 donor-independent metabolites (ADP, Cho, PCho, PCr and U3.48) in osteoblasts differ considerably from those found in hAMSC (Figure S8).

**Fig. 4:**
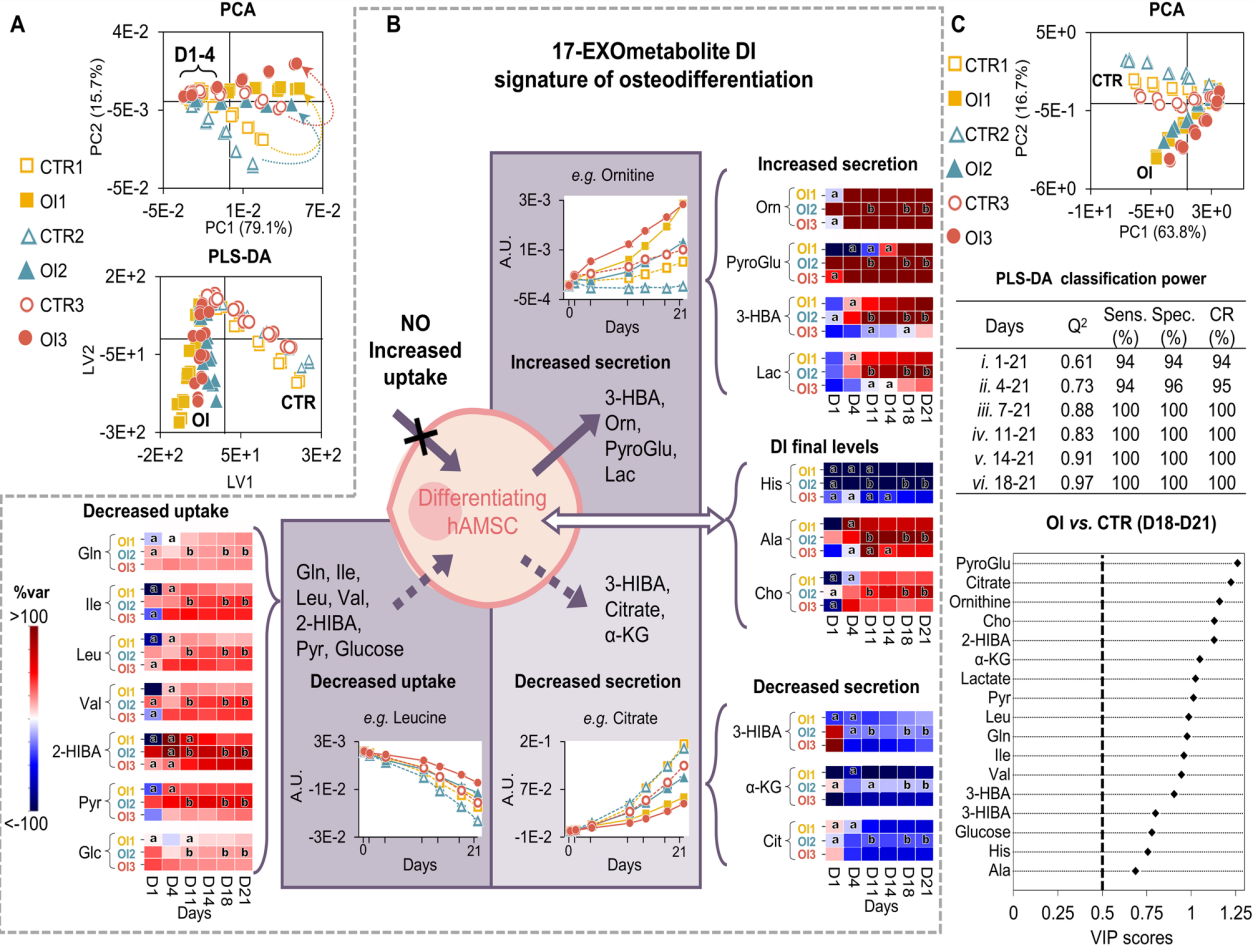
Changes in the exometabolome of osteodifferentiating hAMSC. (**A**) PCA scores scatter plot obtained with the full NMR spectra of the media from hAMSC under control (CTR) and osteoinduced (OI) conditions. (**B**) 17-exometabolite donor-independent (DI) osteogenic signature, which includes patterns of increased secretion (solid arrow, top right), decreased secretion (dashed arrow, bottom right), and reduced uptake (dashed arrow, bottom left) relative to controls, as well as metabolites with donor-dependent patterns but DI final levels (white double arrow). Heatmaps represent % variation in OI compared to CTR, at each timepoint (*p*-values were < 0.05). ^a^ non-significant variation, ^b^*p*-value not computed due to *n* < 3, visual confirmation only. Examples of time-course evolutions are shown in each quadrant (a full account may be found in Figure S9, Additional file 1). (**C**) PCA scores scatter plot obtained only with the 17-exometabolite DI signature (integrals) (top), MCCV results from PLS-DA progressively excluding early timepoints (middle), and VIP scores corresponding to D21 samples. (bottom). Unit variance (UV) used for all multivariate models

The exometabolome profiles of differentiating hAMSC exhibit clear differences compared to controls after D1-D4, as illustrated by PCA and PLS-DA of the full NMR spectra (Fig. 4A). In particular, 17 exometabolites display osteogenic-specific donor-independent time courses (Figure S9A-D, Additional file 1), while the consumption/secretion patterns observed for proliferation alone are qualitatively maintained. Interestingly, no enhanced uptake is noted for any of the detected media metabolites (Fig. 4B, top left). However, differentiating hAMSC require lower amounts of extracellular Gln, BCAA, 2-HIBA, Pyr and Glc, compared to controls (Fig. 4B, bottom left). Conversely, 3-HBA, ornithine (Orn), PyroGlu and Lac are more extensively secreted (Fig. 4B, top right). In addition, 3-HIBA, *α*-KG and Cit (not detected inside the cells) are present in lower amounts in the media of differentiating cells, compared to controls (Fig. 4B, bottom right), which may indicate lesser secretion (and/or a potential specific interaction with the extracellular matrix (ECM), to be discussed below). Within the remaining exometabolites (generally exhibiting donor-dependent behavior), extracellular Ala, Cho and His end-levels are consistent between the 3 donors, compared to controls (Fig. 4B, middle right) and, thus, these metabolites were also considered to be donor-independent. PCA obtained with the integrals of the 17 donor-independent exometabolites effectively distinguishes osteogenic samples from controls (Fig. 4C, top), corroborated by strong PLS-DA models (Q^2^ 0.61–0.97; ≥ 94% sensitivity, specificity and accuracy), particularly after D4 (Fig. 4C, middle). All samples are correctly classified (as controls or osteodifferentiating cells) from D7 onwards, with models iii. to iv. (Fig. 4C, middle) offering promising classification power. PyroGlu, Cit, Orn, Cho and 2-HIBA emerge as the most impacted exometabolites, at D18-D21 (Fig. 4C, bottom). Correlation analysis between endometabolome and exometabolome data (Figure S10) suggests that Orn secretion may relate to decreased intracellular PCr. In addition, the uptake of Asp, Gln, BCAA, Ser and Thr (all anaplerotic substrates), along with secretion of 3-HBA, Cit and Lac, seem closely related to decreased intracellular ATP and ADP. Lastly, extracellular Glu (variable between donors) seems to correlate to increasing levels of UDP-GlcNAc and UDP-GalNAc inside the cells.

## Discussion

### Metabolic adaptations during hAMSC proliferation alone

Changes in the intra- and extracellular metabolic profiles of hAMSC during proliferation alone require consideration when defining specific metabolic markers of differentiation. The donor-independent intracellular signature of proliferation indicates decreased levels of ATP and ADP; increased levels of GPC, HTau, Ino, MG and PEtn; and similar modulation of Asp, BCAA, GSH, PCho and Pro levels. These seem to reflect adaptations in energy metabolism, oxidative stress protection mechanisms, membrane metabolism and Ino metabolism, possibly due to high cell densities and oxygen/nutrient constraints.

Proliferating MSC have been reported to be highly glycolytic and fermentative even in the presence of oxygen (Warburg-like phenomenon), although maintaining a contribution from oxidative phosphorylation (OxPhos) for ATP generation [37]. Different MSC densities determine the precise interplay between glycolysis and OxPhos, with higher cell densities triggering increased glycolysis-to-TCA flux and OxPhos contribution [38]. Our results indicate that both glycolysis and TCA cycle are active. This is shown by high Glc uptake and secretion of Lac (possibly involved in extracellular immunomodulatory signaling [39]), in tandem with uptake of extracellular Pyr, acetate and anaplerotic AA, and secretion of TCA cycle intermediates *α*-KG and Cit. The extensive AA consumption confirms earlier findings [40], although variations previously regarded as donor-independent (for Ala, Glu and Orn) have here been found to be donor-dependent. Similar intracellular modulation of Asp, BCAA and Pro, and secretion of BCAA breakdown products (3-HIBA and 3M2OV) are consistent with tight TCA cycle regulation. Interestingly, BCAA have been suggested to promote MSC proliferation and act as immunomodulatory agents [41]. Secretion of 3-HBA (ketogenesis product of FA *β-*oxidation) may be indicative of FA catabolism, while a signaling role has been suggested for this metabolite in various cell types [42]. Accumulation of extracellular formate has not, to our knowledge, been related before to MSC proliferation but, in other mammalian cells (namely, fibroblasts and selected cancer cells), it has been suggested to arise from mitochondrial serine one-carbon catabolism [43]. The use of ATP (and ADP) seems intricately connected to MG production, to our knowledge newly identified in hAMSC. This is consistent with the observed MG correlations with AA anaplerosis and secretion of TCA intermediates (other MG correlations, *e.g.* with GPC and Ino, may possibly arise from parallel processes). MG has been associated with potential antioxidative roles in both in vitro and in vivo systems [44, 45], possibly arising from creatinine oxidation, mediated by reactive oxygen species (ROS), as a means to mitigate oxidative stress [46]. We hypothesize that uptaken Cr converts into creatinine (not detected) and subsequently to MG, while leaving intracellular Cr levels and Cr/PCr ratios unchanged. Indeed, high-density MSC cultures have been linked to high ROS contents [38], with some MSC therapeutic benefits possibly relating to ROS neutralization capacity [47]. In addition to MG, we suggest that the GSH/GSSG pair is preferentially mobilized for hAMSC oxidative stress protection (decreased GSH levels, with GSSG not detected), leaving the HTau/Tau pair underused for this end, since the reduced form, HTau, increases with time. Decreased GSH may either reflect reduced need for GSH synthesis (low ROS) or enhanced GSH oxidation (elevated ROS). Although ROS levels were not measured in this work, we propose the latter option as more probable in actively proliferating hAMSC cultures. Enhanced GSH synthesis is supported by a positive correlation between cystine uptake and intracellular Glu (which tends to decrease after D1), consistently with the involvement of the cystine/Glu antiporter, as reported in proliferating mouse MSC [48]. Membrane metabolism adaptations are illustrated by increased intracellular PEtn and PCho (after D4) and uptake of extracellular Cho, probably for membrane synthesis. Intracellular GPC accumulation could reflect concomitant phospholipid (PL) hydrolysis, to accommodate some extent of membrane remodeling. The uptake of *m-*Ino may reflect phosphatidylinositols (PtdIno) synthesis (as phosphoinositides generally play a modulatory role in membrane trafficking, *e.g.* vesicle formation) [49], as well as an osmolytic role [50]. Finally, despite the reported significance of extracellular purinergic signaling in MSC proliferation [51], purines were here undetectable in culture media, although intracellular Ino increased with proliferation. Ino may arise from intracellular Ado (not detected or at residual levels) through the action of adenosine deaminase (previously found in hAMSC [51]) and has been reported to have an immunosuppressive effect [51]. The inverse correlation of Ino and ATP suggests that Ado arising from ATP hydrolysis may be rapidly converted into Ino in proliferating hAMSC.

### Metabolic adaptations during hAMSC osteodifferentiation

**Fig. 5:**
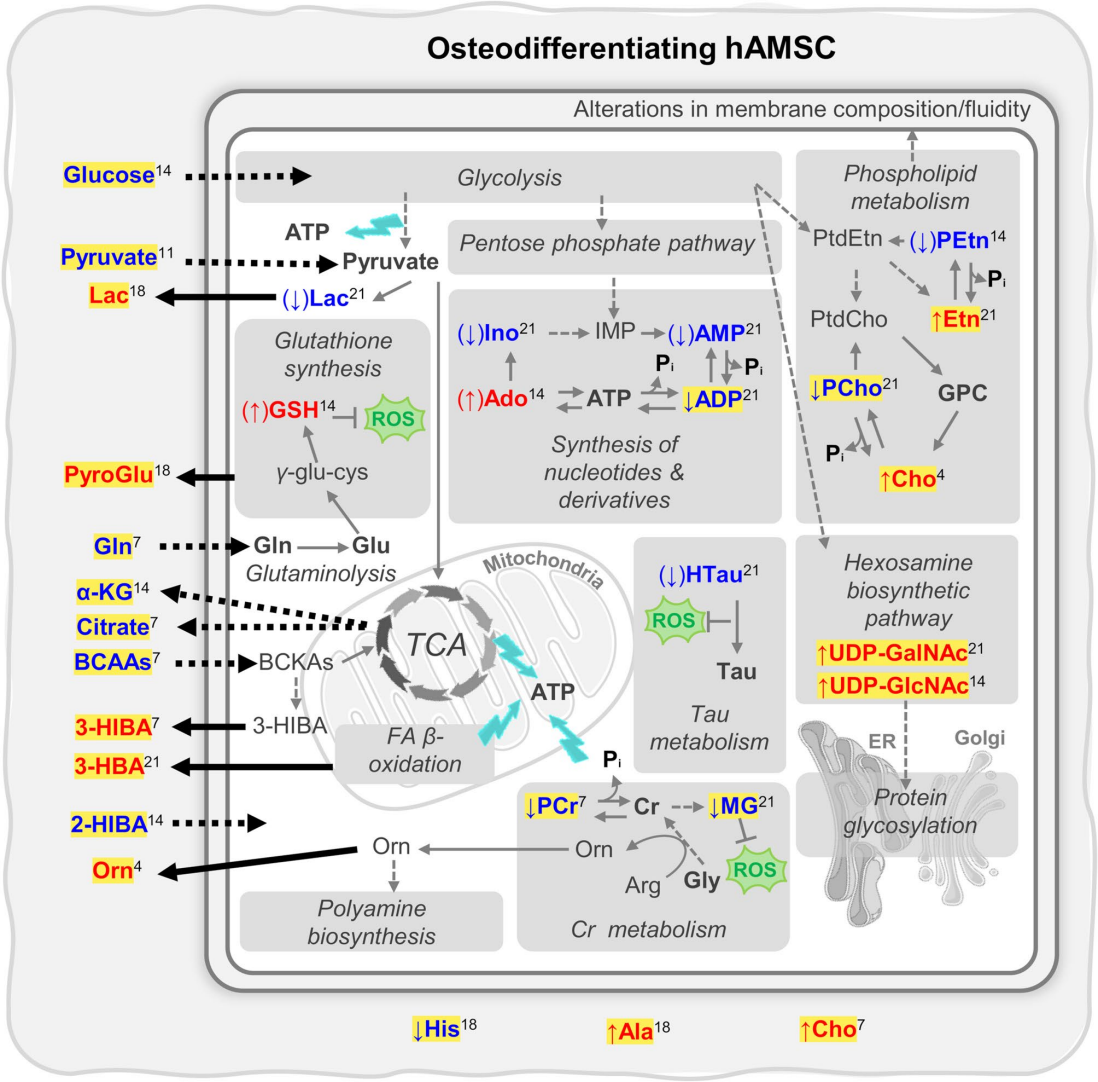
Key metabolic fluctuations in the endo- and exometabolomes of osteodifferentiating hAMSC (compared to proliferation alone). Metabolites detected by NMR are shown in bold. Metabolite names in red and blue were shown to increase or decrease in level, respectively, in osteodifferentiating cells compared to controls (this is also shown by colored arrows next to metabolite names, arrows in brackets reflect variations in 2 of the donors). Superscripts indicate the first day of change. Donor-independent metabolite changes are highlighted in yellow, both inside and outside the cells. Changed metabolite fluxes across the cell membrane are represented by solid or dashed arrows, for enhanced or attenuated fluxes, respectively. Some elements of this picture were adapted from Servier Medical Art (smart.servier.com) and are licensed under a Creative Commons Attribution 3.0 Unported (CC BY 3.0) license. 2-HIBA, 2-hydroxyisobutyrate; 3-HBA, 3-hydroxybutyrate; 3-HIBA, 3-hydroxyisobutyrate; *α*-KG, *α*-ketoglutarate; Ado, adenosine; ADP, adenosine diphosphate; AMP, adenosine monophosphate; ATP, adenosine triphosphate; BCAAs, branched-chained amino acids; BCKAs, branched-chain α-ketoacids; Cho, choline; Cr, creatine; ER, endoplasmic reticulum; FA, fatty acid; GPC, glycerophosphocoline; GSH, glutathione (reduced form); IMP, inosine monophosphate; Ino, inosine; MG, methylguanidine; Orn, ornithine; PCr, phosphocreatine; PEtn, phosphoethanolamine; Pi, inorganic phosphate; PtdCho, phosphatidylcholine; PtdEtn, phosphatidylethanolamine; ROS, reactive oxygen species; TCA, tricarboxylic acid cycle; UDP-GalNAc, uridine diphospho-*N*-acetylgalactosamine; UDP-GlcNAc, uridine diphospho-*N*-acetylglucosamine

A 9-endometabolite intracellular signature (increases in Cho, Etn, UDP-GlcNAc, UDP-GalNAc, U3.48; and decreases in ADP, MG, PCho, PCr) (endometabolites highlighted in yellow in Fig. 5) and a set of altered metabolite ratios (increased Etn/PEtn and Cho/PCho; and decreased HTau/Tau) were found to distinguish osteodifferentiating from proliferating non-differentiating hAMSC, achieving > 90% accuracy as early as D7, and reaching 100% accuracy by D14. Concomitant changes in the secretion/uptake of 17 exometabolites (highlighted in yellow Fig. 5) accurately classified 100% of the samples (as osteodifferentiang or controls) from D7 onwards. The latter signature is expected to enable rapid and non-invasive detection of osteodifferentiation, without the need for cell collection. The absence of increased demand for any of the available exometabolites indicates that osteodifferentiating cells primarily use and redirect existing intracellular resources for the required pathway adaptations. These adaptations notably impact energy metabolism and associated purine and AA pathways, oxidative stress management, membrane metabolism and protein glycosylation.

The usual substrates involved in energy production (Glc, Gln, BCAAs and Pyr) are required to a lesser extent by differentiating cells, compared to proliferating cells. Less Glc uptake may suggest glycolysis downregulation, and the higher secretion of Lac is consistent with Pyr derived from glucose oxidation through glycolysis being preferentially reduced to Lac, rather than oxidized for TCA cycle entry. The resulting lower TCA activity is illustrated by lower Cit and *α*-KG secretion and reduced use of anaplerotic AA (Gln and BCAA). Although it has been generally accepted that OxPhos activity increases during osteodifferentiation, while lactic fermentation decreases (or remains constant) [37, 52, 53], recent metabolic MSC studies have highlighted aerobic glycolysis as an active main energy source [54]. This aligns with our observations of subdued TCA cycle activity (and OxPhos), as well as with previous findings in mature osteoblasts [55]. At a first glance, reduced glucose uptake and TCA activity might suggest that osteodifferentiating cells require less energy. However, we propose that these cells shift from relying primarily on glucose oxidation to also utilizing the PCr-Cr system for ATP production (Fig. 5). This is consistent with the strong direct correlation between intracellular ATP and PCr (*ρ* 0.8) during differentiation. Enhanced conversion of PCr to Cr is shown by increased Cr/PCr ratios after D7, PCr thus serving as a source of ATP and inorganic phosphate (Pi) needed for hydroxyapatite formation. Apart from PCr dephosphorylation, activated Cr biosynthesis (from Arg, Gly and Met) may account for the significantly increased secretion of Orn (Fig. 5), as early as D4 (*p-*value < 0.01 when comparing osteogenic samples from all donors to controls). This could be a mechanism to prevent early inhibition of osteodifferentiation by intracellular polyamines, for which Orn is a precursor [56]. Cr has been shown to promote osteodifferentiation and mineralization of osteoblast-like cells [57], and to improve bone health in humans [58]. However, beyond the involvement of creatine kinase in the mineralization of chicken embryo bone matrix linking Cr to ATP synthesis [59], no other roles for Cr in osteodifferentiation have been suggested, to our knowledge. Additionally, the observed increase in 3-HBA secretion suggests that enhanced FA *β-*oxidation provides supplementary energy (Fig. 5), aligning with previous suggestions that lipids support ECM deposition and mineralization [60]. The proposed downregulation of glycolysis and the TCA cycle may lead to a more efficient management of intracellular resources, to prevent excess nutrients from inhibiting differentiation [61]. Specifically, Glc is expected to be redirected towards collagen synthesis [62], whereas intracellular Lac and TCA intermediates have been suggested to contribute to histone modifications and thus activate osteogenic genes [63, 64]. Furthermore, the increased secretion of Lac may lead to the activation of signaling pathways that modulate immunity [39] and result in more efficient osteodifferentiation [65]. Extracellular Cit could play a role in bone biomineralization by facilitating crystal nucleation and stabilizing hydroxyapatite (HA) nanocrystals [66]. However, the possible entrapment of Cit within the ECM may reduce detectability *via* NMR, hindering its accurate quantitation in cell media.

In addition to the role of purines in ATP generation, we propose that osteodifferentiation triggers a deviation from the predominant Ado-to-Ino conversion (occurring in proliferation) towards higher Ado levels compared to Ino. We suggest that this intracellular Ado accumulation arises from ATP and ADP hydrolysis (Fig. 5), possibly benefitting from the contribution of PCr dephosphorylation to the ATP pool. This is supported by the strong positive correlation between intracellular Ado and Cr levels. Ado has indeed been recognized as having a pro-osteogenic effect, with extracellular Ado promoting mineralization and Runt-Related Transcription Factor 2 (RUNX2) expression *via* A2B adenosine-specific receptors [67]. We hypothesize that intracellular Ado may be transported across the membrane as ATP to regenerate Ado outside the cell. Indeed, ATP secreted during osteodifferentiation has been reported to be dephosphorylated by ectonucleotidases (including ALP), thereby contributing to the extracellular Pi pool required for calcification [68].

While the precise source of ROS during osteodifferentiation is not fully understood, both mitochondrial activity and protein synthesis are probable contributors [69]. As a result, antioxidative mechanisms are needed to prevent ROS-induced disruption of osteodifferentiation [52, 70], leading to a gradual reduction in these toxic by-products over time. Our results emphasize the importance of active antioxidative mechanisms, particularly from D14 onwards, judging by increased GSH levels after D7, and correlation patterns between GSH and Tau. Even though osteodiffrentiation of BMMSC has been proposed to be GSH-independent [52], GSH is generally regarded as important for osteodifferentiation, specifically in preventing the oxidation and degradation of the pro-osteogenic transcription factor RUNX2 protein [69]. Indeed, GSH synthesis was previously found to be fueled by Gln *via* glutaminolysis possibly also impacting lineage commitment [71]. In addition to GSH, we propose that MG and the HTau/Tau pair may also be involved in ROS protection (Fig. 5), in tandem with a possible involvement of Tau in the extracellular signal regulated kinase (ERK) pathway, reported to result in osteodifferentiation enhancement [72].

For the first time to our knowledge, the donor-independent metabolic signature of osteodifferentiation advanced here includes increased intracellular levels of UDP-GlcNAc and UDP-GalNAc (Fig. 5), possibly due to their increased biosynthesis from Glc. These UDP species act as substrates for glycosyltransferases in protein glycosylation, facilitating the attachment of glycans to proteins, either *N*-linked to Asn (GlcNAc residues) and/or *O*-linked to Ser/Thr (GlcNAc or GalNAc residues). Many key proteins in bone formation are believed to be regulated through multiple glycosylation sites [73], their activity depending on the interplay between glycosylation (mainly GlcNAc-based) and phosphorylation. In particular, *O*-GlcNAc modification of lineage-defining transcription factors (*e.g.* RUNX2) modulates the balance between osteogenic and adipogenic lineages in BMMSC [74]. Furthermore, ALP (a glycoprotein) function in osteogenic and adipogenic differentiations of rat MSC has been related to distinct glycosylation patterns [75]. Although limited research has addressed GalNAc-decorated mucin-type *O*-glycoproteins, osteopontin (OPN) and bone sialoprotein (BSP) are well-recognized examples of relevance in bone [76]. Interestingly, in an immortalized hMSC model, inhibiting *O*-glycosylation was found to suppress mineralization, whereas inhibiting *N*-glycosylation enhanced it [77].

Membrane metabolism adaptations during hAMSC osteodifferentiation became apparent through donor-independent intracellular increases in Cho/PCho and Etn/PEtn ratios (reduced levels of PCho and PEtn, and rises in Cho and Etn). These reflect the expected upregulation of PHOSPHO1 phosphatase to produce Pi from PCho and PEtn hydrolysis [78]. The early tendency for intracellular accumulation of PCho and PEtn in donors 1 and 3 (between D1 and D7) suggests that PL degradation may be favored over synthesis, possibly to prepare for subsequent PHOSPHO1 activity. Although this process is commonly believed to take place in extracellular vesicles during mineralization [68], our results suggest that it may be initiated intracellularly.

Although AA are integral to most of the processes discussed above, specific AA behavior was observed in relation to hAMSC osteodifferentiation. The positive Glu-Asp correlation may reflect OPN synthesis, as these AA predominate in this non-collagenous protein [79] which, like OCN, typically peaks late in osteodifferentiation [34]. Furthermore, the higher His levels found here in the media of differentiating cells may reflect its role in regulating HA mineralization and promoting the plate-like morphology found in bone [80]. Finally, extracellular accumulation of PyroGlu in all donors may relate to its role as Glu analogue/reservoir, possibly also contributing towards osmoprotection [81].

As to the assessment of completion of the osteodifferentiation process, it is tempting to compare the metabolic profiles of D21 differentiated hAMSC cells with that of mature osteoblasts, however, this was less than straightforward due to the strong effect of culture times on osteoblast metabolic profiles. It is however interesting to note the absence of MG, Etn, UDP-GlcNAc and UDP-GalNAc (important players in the proposed donor-independent osteogenic signature) in osteoblasts. This could mean that these metabolites are only important during differentiation, with less relevance in fully matured cells and, thus serving as potential markers of ongoing, incomplete, osteodifferentiation. Furthermore, while the remaining osteogenic signature compounds were present in osteoblasts, their relative levels were significantly distinct. This may either be due to differentiated hAMSC retaining some reflection of their original MSC characteristics and/or the specific extent of osteodifferentiation, including the possibility of co-lineage differentiation. These are aspects that warrant further investigation when considering the use of metabolic markers to measure overall osteodifferentiation efficacy.

Finally, it is important to highlight some limitations of this study. Due to the low sensitivity of NMR spectroscopy, a high cell count per sample (3–5 million) was required, resulting in the use of substantial resources and lengthy protocols. In addition, the resulting large dataset (ca. 260 samples) required considerable data processing and analysis. Due to these constraints, the study focused only on three donors (instead of the ideal of five or more), prioritizing a global intra- and extracellular characterization to investigate the metabolic interplay between these environments. This strategy was crucial to facilitate future media-only approaches, which will reduce cell requirements and allow for a scaled-down culture process, if appropriate media-to-cell ratios are maintained. Despite achieving a balance between practicality and scientific rigor, further validation of the proposed metabolic signatures in larger donor cohorts and across multiple passages remains essential. Targeted pathway and enzymatic analyses are also necessary to corroborate/discard the proposed biochemical interpretations. Furthermore, the applicability of the identified markers in MSC from other tissue sources and to various co-lineage differentiation scenarios requires further exploration. The compound resonating at δ 3.48, importantly linked to osteodifferentiation, also remains unidentified. Nonetheless, the current findings highlight the potential of metabolites as reliable indicators of early osteodifferentiation and support the development of a non-invasive, media-based protocols for early donor selection, representing a notable advancement over previous single-donor metabolomic studies.

## Conclusions

We report potential donor-independent intracellular and extracellular metabolite signatures that may be used to detect/predict hAMSC osteodifferentiation by day 7 with nearly 100% accuracy. The intracellular signature comprises increased levels of Cho, Etn, UDP-GlcNAc, UDP-GalNAc and an unknown compound (U3.48); and decreased levels of ADP, MG, PCho, PCr, compared to controls. In addition, the extracellular signature is composed of lower uptake of Gln, BCAA, 2-HIBA, Pyr, Glc; higher secretion of Orn, PytoGlu, 3-HBA, Lac; and lower secretion of 3-HIBA, *α*-KG, Cit; similar osteogenic end levels of Ala, Cho, His compared to controls. These signatures provide exquisite insight into the metabolic features of hAMSC osteodifferentiation and underlying proliferation. Osteodifferentiation is suggested to involve a shift in energy metabolism from glycolysis/OxPhos to enhanced lactic fermentation, FA *β-*oxidation and use of PCr as a source of ATP and Pi. Interestingly, differentiating hAMSC do not require higher uptake of any of the available polar exometabolites, possibly as a result of effective management and redirection of intracellular resources, *e.g.* Glc for collagen synthesis and protein glycosylation, Asp and Glu for OPN synthesis, Lac and TCA intermediates (particularly Cit) to support immunomodulation and biomineralization in the ECM. Distinct phospholipid and GSH/HTau/MG patterns should reflect adaptations in membrane metabolism and antioxidant protection mechanisms, respectively, while Ado accumulation may relate to its reported pro-osteogenic role. An important practical outcome of this work is that the robust extracellular signature found may be measured non-invasively (for instance using a targeted enzymatic tool) and in real time in the cell media of 2D/3D cultures, independently of morphology and without the need for cell harvesting.

This study is not without limitations, namely regarding the clarification of the still unknown compound (U3.48) which is part of the intracellular signature of osteodifferentiation; the requirement for further validation of the proposed maker signatures in a larger donor cohort and across multiple passages; and the need for targeted pathway/enzymatic analysis to corroborate/discard the advanced putative biochemical explanations. Furthermore, the applicability/adaptability of the proposed markers to the osteodifferentiation of other MSC types, and to different extents of co-lineage outcomes, need to be investigated. However, the present results demonstrate the enticing value of metabolites in effectively reporting on the early stages of osteodifferentiation, with a non-invasive protocol based on media analysis being envisaged for early donor selection.

## Electronic supplementary material

Below is the link to the electronic supplementary material.

**Additional File 1**: **Fig. S1**. Biochemical assays obtained for hAMSC under control (CTRi, shaded bars) and osteoinduction conditions (OIi, filled bars) for donors 1 (yellow), 2 (blue), and 3 (red). **Fig. S2.** Average 500 MHz ^1^H NMR spectra of (A) hAMSC polar extracts from donor 1 after 21 days under control conditions (full list of endometabolites can be found in Table S1), (B) blank culture media (uncorrected) and (C) media of proliferating hAMSC from donor 1 at day 21 (corrected; positive and negative peaks indicate secretion and uptake, respectively). **Fig. S3.** PLS-DA scores scatter plots (left) obtained with NMR spectra (UV-scaled) of polar extracts from proliferating hAMSC (controls, CTR) from donors 1 (yellow squares), 2 (blue triangles) and 3 (red circles), and corresponding LV1 loading plots (right) colored according to VIP. **Fig. S4.** Heatmaps of donor-independent exometabolite changes in proliferating hAMSC (% variation compared to D1), for donors 1, 2 and 3 (in yellow, blue, and red, respectively). **Fig. S5.** PLS-DA scores plots (left) and corresponding loadings (right) obtained for 1H NMR spectra (UV-scaled) of polar extracts of osteoinduced hAMSC (OIi, filled symbols) at D7 to D21 and controls (CTRi, open symbols) for (A) donor 1 (yellow squares), (B) donor 2 (blue triangles) and (C) donor 3 (red circles). **Fig. S6.** Time course evolution of donor-independent endometabolite (A) variations and (B) ratios, in osteodifferentiating hAMSC (solid lines, filled symbols) compared to controls (dashed lines, open symbols). **Fig. S7.** Time course evolution of endometabolites and ratios that (A) varied consistently in osteoinduced hAMSC (solid lines, full symbols) from 2 out of 3 donors, and (B) remained identical to controls (dashed lines, open symbols). **Fig. S8.** Bar charts of the relative level of ADP, Cho, PCho, PCr and U3.48 observed in hAMSC at D21 and in osteoblasts (NHOst). **Fig. S9.** Time course evolution of donor-independent exometabolites that distinguish osteodifferentiating hAMSC (solid lines) from controls (dashed lines). **Fig. S10.** Correlation map between endometabolites (y axis) and exometabolites (x axis) (*ρ* >|0.8| and *p-*value < 0.001) throughout hAMSC osteodifferentiation, considering D1, D4, D7, D14 and D21. **Table S1.**^1^H NMR assignment of polar endometabolites identified in hAMSC from each donor under control and/or osteoinductive conditions, and in human osteoblasts (NHOst). **Table S2.** Percentage variation of donor-independent endometabolite signatures for proliferation alone or controls (CTR D0 vs. CTR D21) (top section) and osteodifferentiation (OI D21 vs. CTR D21) (bottom section), along with absolute quantification in the same samples for donor 3 (the only donor for which dsDNA was quantified in the same samples used for metabolomics).

## Data Availability

The dataset supporting the conclusions of this paper is available in the Metabolomics Workbench website (https://www.metabolomicsworkbench.org/). The data is presently identified by the digital object identifier (DOI) 10.21228/M84C2P

## Abbreviations

2-HIBA: 2-hydroxyisobutyrate
2-HIC: 2-hydroxyisocaproate
3-HBA: 3-hydroxybutyrate
3-HIBA: 3-hydroxyisobutyrate
3M2OV: 3-methyl-2-oxovalerate
AA: Amino acids
Ado: Adenosine
ADP: Adenosine diphosphate
ALP: Alkaline phosphatase
ATP: Adenosine triphosphate
AUC: Area under curve
BCAA: Branched-chain AA
Cho: Choline
Cit: Citrate
CR: Classification rate (or accuracy)
Cr: Creatine
CTR: Control
Di: Day i
DMSO: Dimethylsulfoxide
dsDNA: Double-stranded DNA
FKBP5: FK506 binding protein prolyl isomerase 5
Glc: Glucose
GPC: Glycerophosphocholine
GPL: Glycerophospholipids
GSH: Glutathione
hAMSC: Human adipose-derived MSC
hMSC: Human MSC
HSQC: ^1^-^13^ C heteronuclear single quantum correlation
HTau: Hypotaurine
Ino: Inosine
Lac: Lactate
MG: Methylguanidine
m-Ino: m-inositol
MSC: Mesenchymal stem cells
MVA: Multivariate analysis
NHOst: Normal human osteoblasts
NMR: Nuclear magnetic resonance
OI: Osteoinduced
OxPhos: Oxidative phosphorylation
PCA: Principal component analysis
PCho: Phosphocholine
PEtn: Phosphoethanolamine
Pi: Inorganic phosphate
PLS-DA: Partial-least-squares discriminant analysis
Pyr: Pyruvate
PyroGlu: Pyroglutamate
Q^2^: Predictive power
ROC: Receiver operating characteristic
ROS: Reactive oxygen species
RT: Room temperature
RUNX2: Runt-related transcription factor 2
SAMHD1: SAM and HD domain-containing deoxynucleoside triphosphate triphosphohydrolase
sens: Sensitivity
spec: Specificity
Tau: Taurine
TCA: Tricarboxylic acid
TOCSY: Two-dimensional ^1^H-^1^H total correlation
TSP: 3-trimethylsilyl-propionic-2,2,3,3-d4 acid
UDP-GalNAc: Diphospho-N-acetylgalactosamine
UDP-GlcNAc: Uridine diphospho-N-acetylglucosamine
UV: Unit variance
VIP: Variable importance to the projection
*α*-KG: *α*-ketoglutarate
*β*-GP: *β*-glycerophosphate
