# Supplementary material for: Metabolic markers detect early ostedifferentiation of mesenchymal stem cells from multiple donors
Source: Stem Cell Res Ther. 2025 Jun 7;16:294. doi: 10.1186/s13287-025-04419-x (PMC12145603; doi:10.1186/s13287-025-04419-x)
Supplement: Supplementary file 1 — Additional File 1: Fig. S1. Biochemical assays obtained for hAMSC under control (CTRi, shaded bars) and osteoinduction conditions (OIi, filled bars) for donors 1 (yellow), 2 (blue), and 3 (red). Fig. S2. Average 500 MHz 1H NMR spectra of (A) hAMSC polar extracts from donor 1 after 21 days under control conditions (full list of endometabolites can be found in Table S1), (B) blank culture media (uncorrected) and (C) media of proliferating hAMSC from donor 1 at day 21 (corrected; positive and negative peaks indicate secretion and uptake, respectively). Fig. S3. PLS-DA scores scatter plots (left) obtained with NMR spectra (UV-scaled) of polar extracts from proliferating hAMSC (controls, CTR) from donors 1 (yellow squares), 2 (blue triangles) and 3 (red circles), and corresponding LV1 loading plots (right) colored according to VIP. Fig. S4. Heatmaps of donor-independent exometabolite changes in proliferating hAMSC (% variation compared to D1), for donors 1, 2 and 3 (in yellow, blue, and red, respectively). Fig. S5. PLS-DA scores plots (left) and corresponding loadings (right) obtained for 1H NMR spectra (UV-scaled) of polar extracts of osteoinduced hAMSC (OIi, filled symbols) at D7 to D21 and controls (CTRi, open symbols) for (A) donor 1 (yellow squares), (B) donor 2 (blue triangles) and (C) donor 3 (red circles). Fig. S6. Time course evolution of donor-independent endometabolite (A) variations and (B) ratios, in osteodifferentiating hAMSC (solid lines, filled symbols) compared to controls (dashed lines, open symbols). Fig. S7. Time course evolution of endometabolites and ratios that (A) varied consistently in osteoinduced hAMSC (solid lines, full symbols) from 2 out of 3 donors, and (B) remained identical to controls (dashed lines, open symbols). Fig. S8. Bar charts of the relative level of ADP, Cho, PCho, PCr and U3.48 observed in hAMSC at D21 and in osteoblasts (NHOst). Fig. S9. Time course evolution of donor-independent exometabolites that distinguish osteodifferenti [file 13287_2025_4419_MOESM1_ESM.docx]

Additional File 1 to manuscript:

Metabolic markers detect early ostedifferentiation of

mesenchymal stem cells from multiple donors

Daniela S. C. Bispo^1^, Inês C. R. Graça^1^, Catarina S. H. Jesus^1^, João E. Rodrigues^1^, Marlene C. Correia^1^, Sabrina Atella^1^, Iola F. Duarte^1^, Brian J. Goodfellow^1^, Mariana B. Oliveira^1^, João F. Mano^1,^**, Ana M. Gil^1,^*

^1^ Department of Chemistry, CICECO - Aveiro Institute of Materials (CICECO/UA), University of Aveiro, Campus Universitario de Santiago, 3810-193 Aveiro, Portugal

* Correspondence: agil@ua.pt; Tel.: +351 234370707, jmano@ua.pt: Tel.: +351 234370733

**This file includes:**

**Fig. S1.** Biochemical assays obtained for hAMSC under control (CTRi, shaded bars) and osteoinduction conditions (OIi, filled bars) for donors 1 (yellow), 2 (blue), and 3 (red).

**Fig. S2.** Average 500 MHz ^1^H NMR spectra of (A) hAMSC polar extracts from donor 1 after 21 days under control conditions (full list of endometabolites can be found in Table S1), (B) blank culture media (uncorrected) and (C) media of proliferating hAMSC from donor 1 at day 21 (corrected; positive and negative peaks indicate secretion and uptake, respectively).

**Fig. S3.** PLS-DA scores scatter plots (left) obtained with NMR spectra (UV-scaled) of polar extracts from proliferating hAMSC (controls, CTR) from donors 1 (yellow squares), 2 (blue triangles) and 3 (red circles), and corresponding LV1 loading plots (right) colored according to VIP.

**Fig. S~~4~~.** Heatmaps of donor-independent exometabolite changes in proliferating hAMSC (% variation compared to D1), for donors 1, 2 and 3 (in yellow, blue, and red, respectively).

**Fig. S5.** PLS-DA scores plots (left) and corresponding loadings (right) obtained for ^1^H NMR spectra (UV-scaled) of polar extracts of osteoinduced hAMSC (OIi, filled symbols) at D7 to D21 and controls (CTRi, open symbols) for (A) donor 1 (yellow squares), (B) donor 2 (blue triangles) and (C) donor 3 (red circles).

**Fig. S6.** Time course evolution of donor-independent endometabolite (A) variations and (B) ratios, in osteodifferentiating hAMSC (solid lines, filled symbols) compared to controls (dashed lines, open symbols).

**Fig. S7.** Time course evolution of endometabolites and ratios that (A) varied consistently in osteoinduced hAMSC (solid lines, full symbols) from 2 out of 3 donors, and (B) remained identical to controls (dashed lines, open symbols).

**Fig. S8.** Bar charts of the relative level of ADP, Cho, PCho, PCr and U3.48 observed in hAMSC at D21 and in osteoblasts (NHOst).

**Fig. S9.** Time course evolution of donor-independent exometabolites that distinguish osteodifferentiating hAMSC (solid lines) from controls (dashed lines).

**Fig. S10.** Correlation map between endometabolites (y axis) and exometabolites (x axis) (*ρ* > |0.8| and *p-*value < 0.001) throughout hAMSC osteodifferentiation, considering D1, D4, D7, D14 and D21.

**Table S1.** ^1^H NMR assignment of polar endometabolites identified in hAMSC from each donor under control and/or osteoinductive conditions, and in human osteoblasts (NHOst).

**Table S2.** Percentage variation of donor-independent endometabolite signatures for proliferation alone or controls (CTR D0 *vs*. CTR D21) (top section) and osteodifferentiation (OI D21 *vs*. CTR D21) (bottom section), along with absolute quantification in the same samples for donor 3 (the only donor for which dsDNA was quantified in the same samples used for metabolomics).

**

Figure S1**

Fig. S1. Biochemical assays obtained for hAMSC under control (CTRi, shaded bars) and osteoinduction conditions (OIi, filled bars) for donors 1 (yellow), 2 (blue), and 3 (red). (A) dsDNA levels, (B) alkaline phosphatase (ALP) activity, (C) calcium ion and (D) osteocalcin (OCN) contents, normalized to dsDNA. Data shown as mean ± standard deviation. *, *p-*values < 0.05; **, *p-*values < 0.01; ^(^*^)^, *p-*values < 0.09 (applicable when *n* = 2 in one of the groups).

**Figure S2**





Fig. S2. Average 500 MHz ^1^H NMR spectra of (A) hAMSC polar extracts from donor 1 after 21 days under control conditions (full list of endometabolites can be found in Table S1), (B) blank culture media (uncorrected) and (C) media of proliferating hAMSC from donor 1 at day 21 (corrected; positive and negative peaks indicate secretion and uptake, respectively). Peak assignments: 1. Ile, 2. Leu, 3. Val, 4. propionate, 5. propylene glycol (contaminant), 6. Lactate (Lac) 7. Thr, 8. Ala, 9. Lys, 10. Arg, 11. acetate, 12. Pro, 13. Glu, 14. uridine diphospho-*N*-acetylglucosamine (UDP-GlcNAc), 15. Gln, 16. glutathione (reduced) (GSH), 17. acetone, 18. succinate, 19. hypotaurine (HTau), 20. Asp, 21. methylguanidine (MG), 22. Asn, 23. creatine, 24. phosphocreatine, 25. ethanolamine (Etn), 26. choline (Cho), 27. phosphoethanolamine, 28. phosphocholine (PCho), 29. glycerophosphocholine, 30. Tau, 31. *m*-inositol, 32. Gly, 33. glucose, 34. uridine diphospho-*N*-acetylgalactosamine (UDP-GalNAc), 35. uracil, 36. uridine, 37. guanosine, 38. nicotinamide adenine dinucleotide (NAD^+^), 39. adenosine, 40. inosine, 41. adenosine diphosphate (ADP), 42. adenosine triphosphate (ATP), 43. adenosine monophosphate (AMP), 44. Tyr, 45. His, 46. Phe, 47. formate, 48. 2-hydroxyisobutyrate (2-HIBA), 49. Pyroglutamate (PyroGlu), 50. Met, 51. pyruvate, 52. cystine, 53. Fructose, 54. Ser, 55. Trp, 56. 3-methyl-2-oxovalerate (3M2OV), 57. 3-hydroxyisobutyrate (3-HIBA), 58. 3- hydroxybutyrate (3-HBA), 59. Citrate (Cit). *, excluded spectral regions.

Figure S3



Fig. S3. PLS-DA scores scatter plots (left) obtained with NMR spectra (UV-scaled) of polar extracts from proliferating hAMSC (controls, CTR) from donors 1 (yellow squares), 2 (blue triangles) and 3 (red circles), and corresponding LV1 loading plots (right) colored according to VIP. Pairwise comparison of (A) donors 1 and 2, (B) donors 1 and 3 and (C) donors 2 and 3. Metabolites abbreviations as defined in the caption of Table S1; Dj, day j; LV: latent variable; Q^2^: predictive power; Uδ: unassigned signal at chemical shift δ.

Figure S4


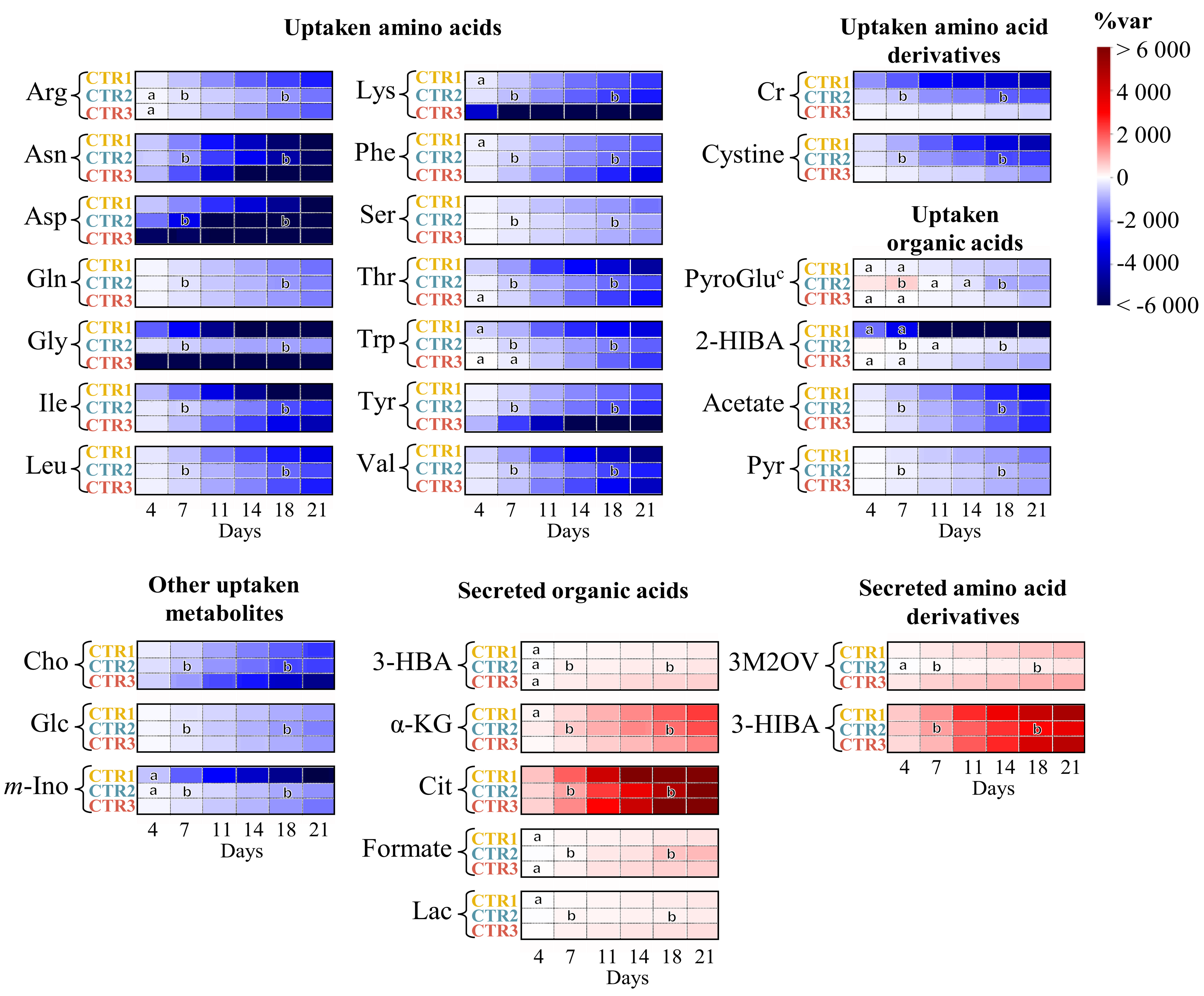


Fig. S4. Heatmaps of donor-independent exometabolite changes in proliferating hAMSC (% variation compared to D1), for donors 1, 2 and 3 (in yellow, blue, and red, respectively). Abbreviations as defined in the caption of Table S1 and Figure S2, and three-letter code for amino acids. All *p*-values < 0.05 except for (^a^) qualitative variations and (^b^) visually confirmed changes for which *p*-values could not be computed due to n < 3.

Figure S5





Fig. S5. PLS-DA scores plots (left) and corresponding loadings (right) obtained for ^1^H NMR spectra (UV-scaled) of polar extracts of osteoinduced hAMSC (OIi, filled symbols) at D7 to D21 and controls (CTRi, open symbols) for (A) donor 1 (yellow squares), (B) donor 2 (blue triangles) and (C) donor 3 (red circles). Metabolite abbreviations as defined in the caption of Table S1. LV: latent variable; Q^2^: predictive power; Uδ: unassigned signal at chemical shift δ.

Figure S6





Fig. S6. Time course evolution of donor-independent endometabolite (A) variations and (B) ratios, in osteodifferentiating hAMSC (solid lines, filled symbols) compared to controls (dashed lines, open symbols). Yellow bars: statistically significant differences between osteodifferentiating and control cells (*p*-value < 0.05), except for D21 from donor 2 (visual inspection as *n* = 1 for OI2). Metabolite abbreviations specified in Table S1. Di, day i; Uδ, unassigned signal at δ. *, *p-*values < 0.05 (compared to previous timepoint); ^(^*^)^, visual changes (n < 3).



Figure S7

Fig. S7. Time course evolution of endometabolites and ratios that (A) varied consistently in osteoinduced hAMSC (solid lines, full symbols) from 2 out of 3 donors, and (B) remained identical to controls (dashed lines, open symbols). Yellow bars: statistically significant differences between OI and CTR (*p*-value < 0.05) for each time point, except for D21 from donor 2 (visual inspection as *n* = 1 for OI2). Metabolite abbreviations specified in the caption of Table S1. Di, day i; Uδ, unassigned signal at δ. *, *p-*values < 0.05 (compared to previous timepoint); ^(^*^)^, visual changes only, as n < 3.

**Figure S8**



Fig. S8. Bar charts of the relative level of ADP, Cho, PCho, PCr and U3.48 observed in hAMSC at D21 and in osteoblasts (NHOst). Osteoinduced hAMSC (filled bars), control hAMSC (dashed bars) from donors 1 (yellow), 2 (blue) and 3 (red), and NHOst culture time 6 days (black) and 13 days (grey).

Figure S9





Fig. S9. Time course evolution of donor-independent exometabolites that distinguish osteodifferentiating hAMSC (solid lines) from controls (dashed lines). This includes exometabolites that show relatively (A) increased secretion, (B) decreased secretion, (C) decreased uptake and (D) variable changes with donor-independent final levels. Metabolite abbreviations as specified in the caption of Figure S2.

Figure S10





Fig. S10. Correlation map between endometabolites (y axis) and exometabolites (x axis) (*ρ* > |0.8| and *p-*value < 0.001) throughout hAMSC osteodifferentiation, considering D1, D4, D7, D14 and D21. Metabolite abbreviations as defined in the caption of Table S1 and Figure S2.

**Table S1. ^1^H NMR assignment of polar endometabolites identified in hAMSC from each donor under control and/or osteoinductive conditions, and in human osteoblasts (NHOst).** Metabolite ID numbers from the Human Metabolome Database (HMDB) and the Kyoto Encyclopedia of Genes and Genomes (KEGG) database are listed. Abbreviations: 1-MNA, 1-methyl-nicotinamide; 2-HIC, 2-hydroxyisocaproate; Ado, adenosine; ADP, adenosine diphosphate; AMP, adenosine monophosphate; ATP, adenosine triphosphate; Cho, choline; Cr, creatine; Etn, ethanolamine; Glc, glucose; GPC, glycerophosphocholine; GSH, glutathione reduced; HTau, typotaurine; Ino, inosine; Lac, lactate; MG, methylguanidine; *m*-Ino, *m*-inositol; PCho, phosphocholine; PCr, phosphocreatine; PEtn, phosphoethanolamine; Pyr, pyruvate. Three-letter code used for amino acids. s, singlet; d, doublet; dd, doublet of doublets; dt, doublet of triplets; t, triplet; q, quartet; m, multiplet. Symbols: ^a^, Peak used for integration (part of spin system); ^b^, tentative assignment; ^c^, identified for the first time in hAMSC, to the best of our knowledge; ^d^, possible contaminant; **✓**, metabolite clearly detected; (**✓**), metabolite detected close to noise level; ✗, metabolite not detected.

| Metabolite | HMDB ID/KEGG ID | *δ* ^1^H in ppm (multiplicity, assignment) ^a^ | hAMSC | | | | | NHOst |
| --- | --- | --- | --- | --- | --- | --- | --- | --- |
|  |  |  | Donor 1 | Donor 2 | | | Donor 3 |  |
| *Amino Acids and Derivatives* | | | | | | | | |
| Ala | HMDB0000161/C00041 | 1.48 (d, *β-*C**H_3_**) | **✓** | **✓** | | | **✓** | **✓** |
| Asn | HMDB0000168/C00152 | - | (**✓**) | (**✓**) | | | ✗ | ✗ |
| Asp | HMDB0000191/C00049 | 2.82 (dd, *β-*C**H_2_**) | **✓** | **✓** | | | **✓** | **✓** |
| Glu | HMDB0000148/C00025 | 2.35 (m, *γ-*C**H**_2_) | **✓** | **✓** | | | **✓** | **✓** |
| Gln | HMDB0000641/C00064 | 2.46 (m, *γ-*C**H_2_**) | **✓** | (**✓**) | | | **✓** | **✓** |
| Gly | HMDB0000123/C00037 | 3.56 (s, α-C**H_2_**) | **✓** | **✓** | | | **✓** | **✓** |
| His | HMDB0000177/C00135 | 7.08 (s, 5-C**H** ring) | (**✓**) | (**✓**) | | | ✗ | ✗ |
| Ile | HMDB0000172/C00407 | 1.02 (d, *γ’*-C**H_3_**) | **✓** | **✓** | | | **✓** | **✓** |
| Leu | HMDB0000687/C00123 | 0.97 (d, *δ*-C**H_3_**) | **✓** | **✓** | | | **✓** | **✓** |
| Lys | HMDB0000182/C00047 | 3.03 (t, *ε*-C**H_2_**, t) | **✓** | **✓** | | | (**✓**) | (**✓**) |
| Met | HMDB0000696/C00073 | - | (**✓**) | (**✓**) | | | ✗ | ✗ |
| Phe | HMDB0000159/C00079 | 7.34 (m, 2-C**H**  & 6-C**H** ring) | **✓** | **✓** | | | (**✓**) | ✗ |
| Pro | HMDB0000162/C00148 | 2.04 (m, *γ*-C**H_2_**) | **✓** | **✓** | | | (**✓**) | ✗ |
| Tau | HMDB0000251/C00245 | 3.42 (t, N-C**H_2_**) | **✓** | **✓** | | | **✓** | **✓** |
| Thr | HMDB0000167/C00188 | 3.59 (d, *β*-C**H)** | (**✓**) | (**✓**) | | | ✗ | ✗ |
| Tyr | HMDB0000158/C00082 | 7.20 (d, 2-C**H**  & 6-**H** ring) | **✓** | **✓** | | | (**✓**) | ✗ |
| Val | HMDB0000883/C00183 | 1.00 (d, *γ’*-C**H_3_**) | **✓** | **✓** | | | **✓** | **✓** |
| Cr | HMDB0000064/C00300 | 3.04 (s, N-C**H_3_**) | **✓** | **✓** | | | **✓** | **✓** |
| GSH | HMDB0000125/C00051 | 2.96 (m, *β*-C**H_2_** Cys) | **✓** | **✓** | | | (**✓**) | (**✓**) |
| HTau ^b,c^ | HMDB0000965/C00519 | 2.65 (t, *β*-C**H_2_**) | **✓** | (**✓**) | | | (**✓**) | ✗ |
| MG ^b^ | HMDB0001522/C02294 | 2.85 (s, C**H_3_**) | **✓** | **✓** | | | **✓** | ✗ |
| PCr | HMDB0001511/C02305 | 3.05 (s, N-C**H_3_**) | **✓** | **✓** | | | **✓** | **✓** |
| *Membrane precursors* | | | | | | | | |
| Cho | HMDB0000097/C00114 | 3.21 (s, N(C**H_3_**)_3_) | **✓** | **✓** | | | **✓** | **✓** |
| Etn | HMDB0000149/C00189 | 3.14 (t, C**H_2_**-NH_2_) | **✓** | **✓** | | | (**✓**) | ✗ |
| GPC | HMDB0000086/C00670 | 3.24 (s, N(C**H_3_**)_3_) | **✓** | **✓** | | | **✓** | **✓** |
| PCho | HMDB0001565/C00588 | 3.23 (s, N(C**H_3_**)_3_) | **✓** | **✓** | | | **✓** | **✓** |
| PEtn ^b^ | HMDB0000224/C00346 | 3.22 (m, N-C**H_2_**) | **✓** | **✓** | | | (**✓**) | **✓** |
| *Nucleotides and derivatives* | | | | | | | | |
| Ado | HMDB0000050/C00212 | 8.27 (s, 2-CH ring) | **✓** | **✓** | | | (**✓**) | ✗ |
| ADP | HMDB0001341/C00008 | 8.54 (s, 8-C**H** ring) | **✓** | **✓** | | | **✓** | **✓** |
| AMP | HMDB0000045/C00020 | 8.62 (s, 8-C**H** ring) | **✓** | **✓** | | | (**✓**) | ✗ |
| ATP | HMDB0000045/C00020 | 8.55 (s, 8-C**H** ring) | **✓** | **✓** | | | **✓** | **✓** |
| Guanosine ^b^ | HMDB0000133/C00387 | 8.01 (s, 8-C**H** ring) | **✓** | **✓** | | | ✗ | ✗ |
| Ino | HMDB0000195/C00294 | 8.24 (s, 2-C**H** ring) | **✓** | **✓** | | | **✓** | **✓** |
| 1-MNA | HMDB0003152/C02918 | - | (**✓**) | ✗ | | | ✗ | ✗ |
| NAD^+^ | HMDB0000902/C00003 | 8.43 (s, 8-C**H** adenine) | **✓** | **✓** | | | (**✓**) | (**✓**) |
| UDP-GalNAc | HMDB0000304/G10611 | 5.55 (dd, 1’’-C**H** galactose) | **✓** | (**✓**) | | | (**✓**) | ✗ |
| UDP-GlcNAc | HMDB0000290/C00043 | 5.52 (dd, 1’’-C**H** glucose) | **✓** | **✓** | | | **✓** | ✗ |
| Uridine | HMDB0000296/C00299 | 7.88 (d, 6-C**H** ring) | **✓** | **✓** | | | ✗ | ✗ |
| Uracil | HMDB0000300/C00106 | - | (**✓**) | ✗ | | | ✗ | ✗ |
| *Organic acids* | | | | | | | | |
| Acetate | HMDB0000042/C00033 | 1.92 (s, *β-*C**H_3_**) | **✓** | **✓** | | | **✓** | **✓** |
| Formate | HMDB0000190/C00186 | 8.46 (s, HO-**H**C=O) | **✓** | **✓** | | | **✓** | **✓** |
| 2-HIC ^c^ | HMDB0000746, - | - | ✗ | ✗ | | | (**✓**) | ✗ |
| Lac | HMDB0000190/C00186 | 4.11 (q, C**H**) | **✓** | **✓** | | | **✓** | **✓** |
| Propionate | HMDB0000237/C00163 | 1.06 (s, C**H_3_**) | **✓** | **✓** | | | ✗ | ✗ |
| Pyr | HMDB0000243/C00022 | 2.38 (s, C**H_3_**) | (**✓**) | (**✓**) | | | ✗ | ✗ |
| Succinate | HMDB0000254/C00042 | 2.41 (s, C**H_2_**) | **✓** | **✓** | | | (**✓**) | (**✓**) |
| *Other metabolites* | | | | | | | | |
| Acetone | HMDB0001659/C00207 | 2.24 (s, *α*-C**H_3_**) | **✓** | **✓** | | | **✓** | **✓** |
| Glc | HMDB0003345/C00031 | 5.24 (d, 1-C**H**) | **✓** | **✓** | | | **✓** | ✗ |
| Glycerol | HMDB0000131/C00116 | 3.56 (dd, 1-C**H_2_**  & 3-C**H_2_**) | ✗ | **✓** | | | **✓** | ✗ |
| *m*-Ino | HMDB0000211/C00137 | 3. 63 (t, 4-C**H**  & 6-C**H**) | **✓** | **✓** | | | **✓** | **✓** |
| Propylene glycol ^d^ | HMDB0001881/C02912 | 1.15 (d, C**H_3_**) | **✓** | **✓** | | | ✗ | ✗ |
|  |  |  |  | |  |  | |  |

**Table S2. Percentage variation of donor-independent endometabolite signatures for proliferation alone or controls (CTR D0 *vs*. CTR D21) (top section) and osteodifferentiation (OI D21 *vs*. CTR D21) (bottom section), along with absolute quantification in the same samples for donor 3 (the only donor for which dsDNA was quantified in the same samples used for metabolomics).** Metabolite abbreviations as defined in the caption of Table S1. *, *p-*values < 0.05; ^(^*^)^, *p-*values < 0.09 (applicable when *n* = 2 samples in one of the groups); ^a^, not found in the Chenomx database and hence not quantified; ND, not detected.

| Metabolite with a donor-independent variation | % variation  (Donor 1 / Donor 2 / Donor 3) | Concentration (donor 3)  (μM/μg dsDNA) | |
| --- | --- | --- | --- |
|  |  |  |  |
| *Proliferation* | ***CTR D0 vs. CTR D21*** | ***CTR3 D0*** | ***CTR3 D21*** |
| HTau | 550 ^(^*^)^ / 101* / 168* | - ^a^ | - ^a^ |
| MG | 241 ^(^*^)^/ 85*/ 200* | 0.1 ± 0.1 | 1.0 ± 0.1 |
| GPC | 177 ^(^*^)^/ 131*/ 190* | 0.3 ± 0.2 | 1.8 ± 0.5 |
| PEtn | 190^(^*^)^/ 152*/ 235 | ND | 5.6 ± 1.6 |
| ADP | -65^(^*^)^/ -56*/ -59 | 1.2 ± 0.7 | 1.2 ± 0.3 |
| ATP | -63^(^*^)^/ -73*/ -56 | 1.0 ± 0.8 | 0.9 ± 0.5 |
| Ino | 161^(^*^)^/ 368*/ 265* | ND | 1.6 ± 0.2 |
| *Osteodifferentiation* | ***OI D21 vs. CTR D21*** | ***CTR3 D21*** | ***OI3 D21*** |
| MG | -34* / -32 ^(^*^)^ / -27* | 1.0 ± 0.1 | 0.7 ± 0.1 |
| PCr | -45* / -68^(^*^)^ / -51* | 0.6 ± 0.0 | 0.3 ± 0.2 |
| Cho | 206* / 172^(^*^)^ / 287* | 0.3 ± 0.1 | 1.6 ± 0.3 |
| Etn | 83* / 146^(^*^)^ / 475* | ND | 2.2 ± 0.4 |
| PCho | -46* / -83 ^(^*^)^ / -45* | 0.4 ± 0.1 | 0.3 ± 0.2 |
| ADP | -48* / -50 ^(^*^)^ / -64* | 1.2 ± 0.3 | ND |
| UDP-GalNAc | 378* / 3418^(^*^)^ / 893* | - ^a^ | - ^a^ |
| UDP-GlcNAc | 970* / 1766^(^*^)^ / 727* | 1.1 ± 1.1 | 6.2 ± 1.2 |
| U3.48 | 149* / 239^(^*^)^ / 974* | - ^a^ | - ^a^ |
